# Supplementary figures and images for: Reproducibility across single-cell RNA-seq protocols for spatial ordering analysis
Source: PLoS One. 2020 Sep 28;15(9):e0239711. doi: 10.1371/journal.pone.0239711 (PMC7521718; doi:10.1371/journal.pone.0239711)

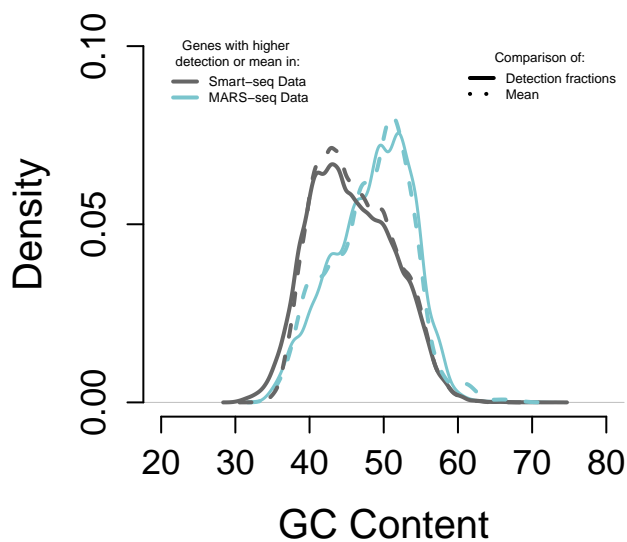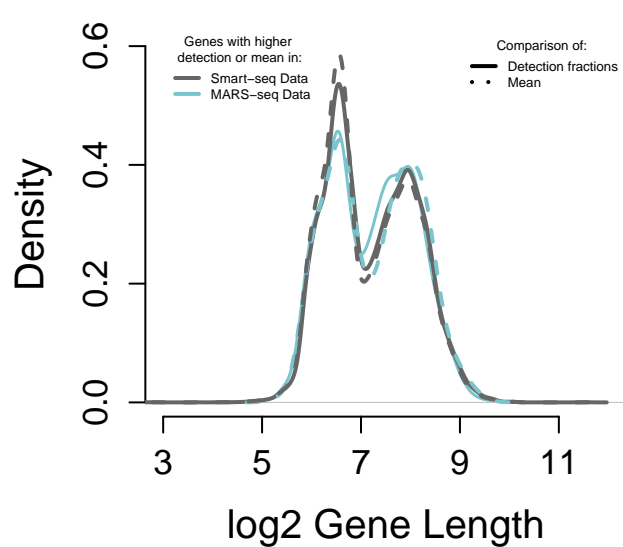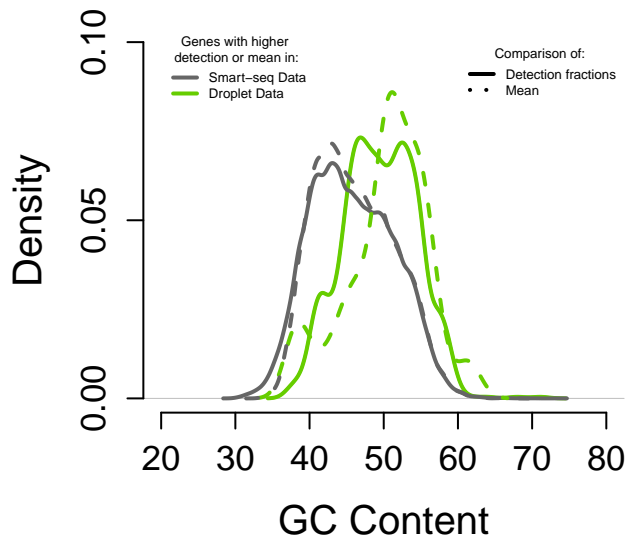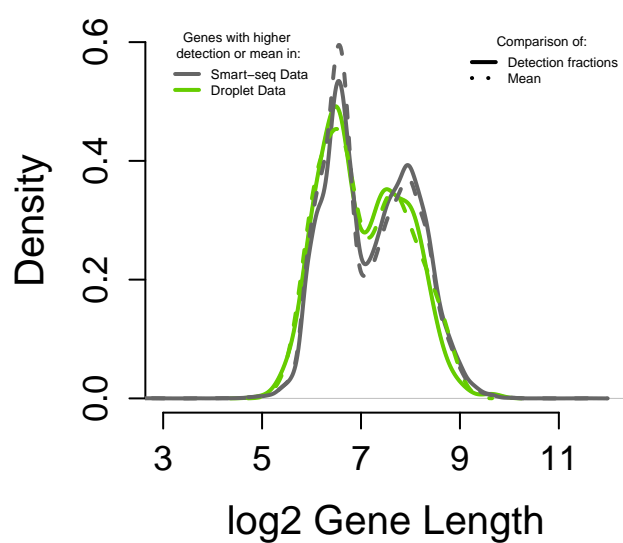

Supplement: S1 Fig — Top) The GC content (left) and gene length (right) are shown for genes having a higher detection fraction in either the Smart-seq dataset (gray) or the MARS-seq dataset (blue). A dotted line is shown for genes having a larger mean in either dataset. The two lines closely correspond since the genes having a high detection fraction typically have a higher mean. Bottom) Similar to the top for comparing the Smart-seq and 10X datasets. (PDF) [file pone.0239711.s001.pdf]

# Cell ordering

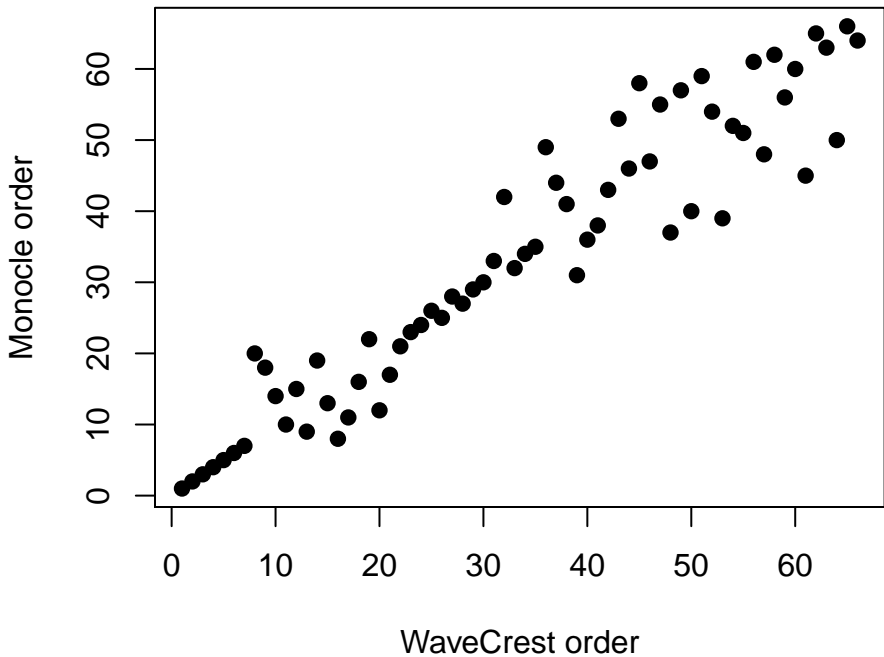

Supplement: S2 Fig — (PDF) [file pone.0239711.s002.pdf]

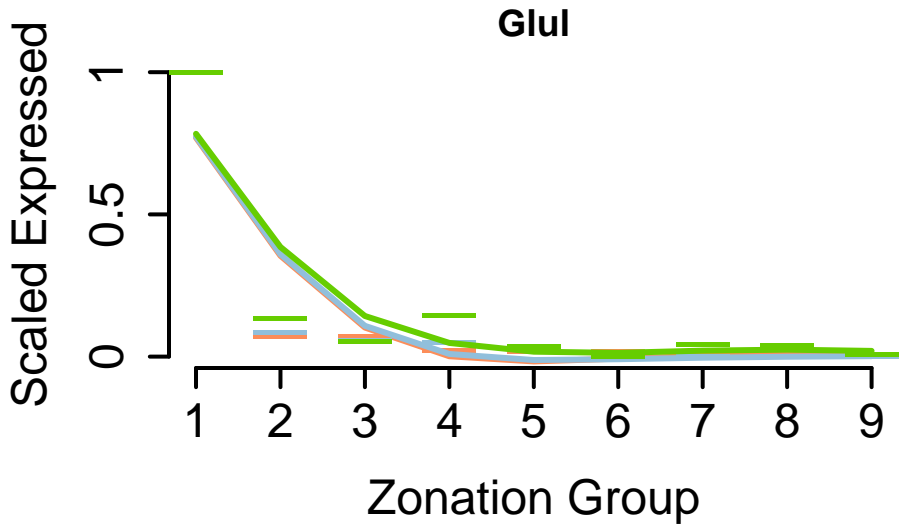

Supplement: S3 Fig — Scaled expression plots of Glul showing high correlation among all three datasets. (PDF) [file pone.0239711.s003.pdf]

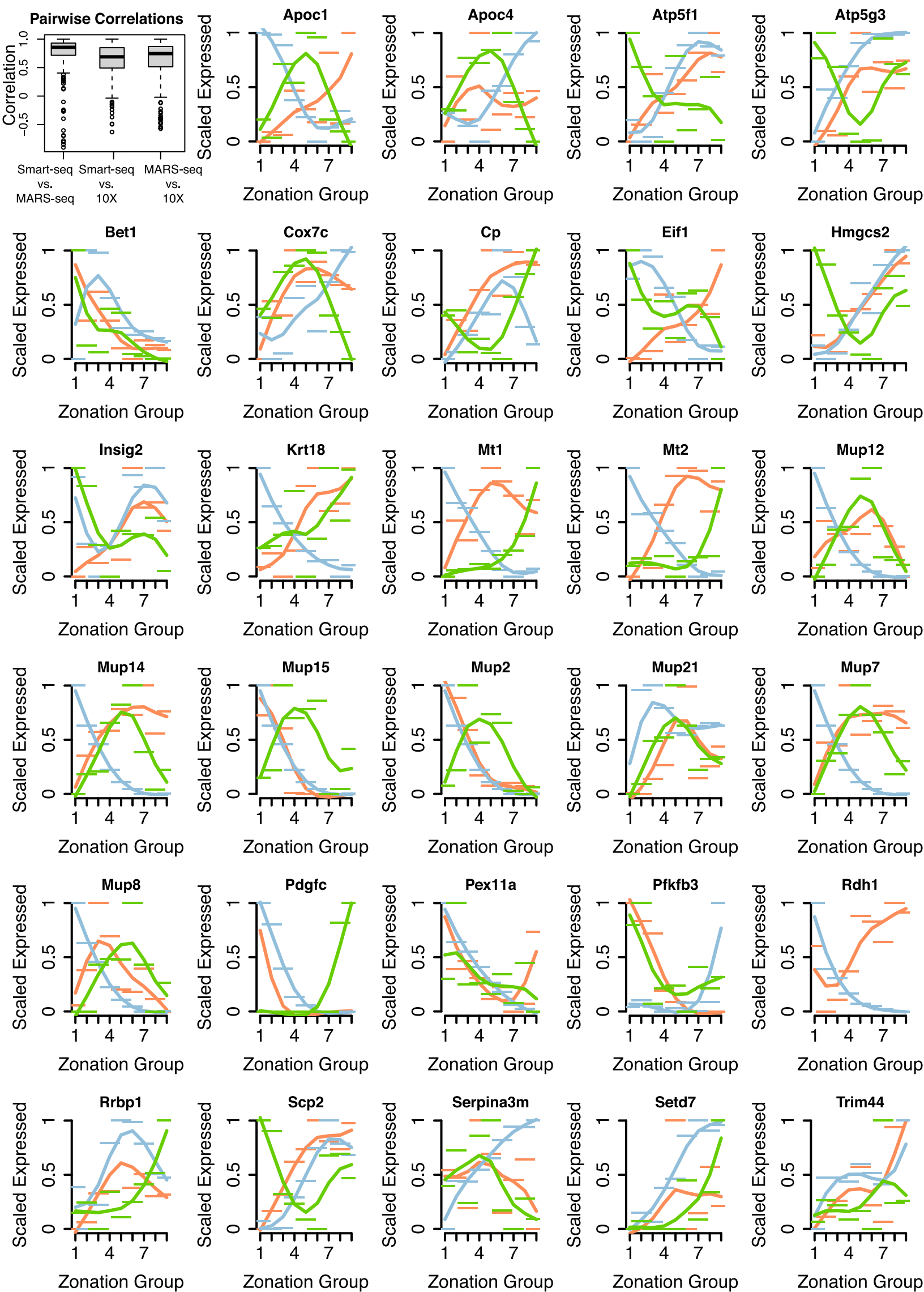

Supplement: S4 Fig — Top left: Pairwise correlations of the expression profiles of all significantly zonated genes. Scatter plots are shown for all genes with correlation less than zero in at least one pairwise correlation. (PDF) [file pone.0239711.s004.pdf]

**A**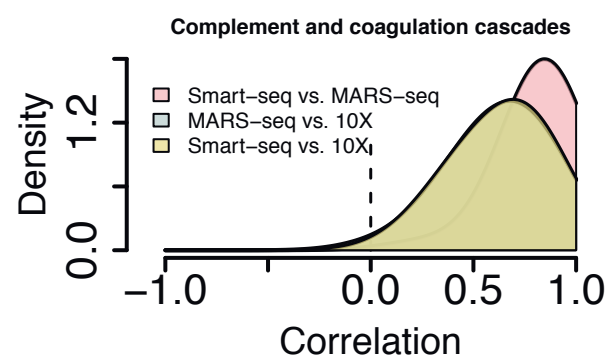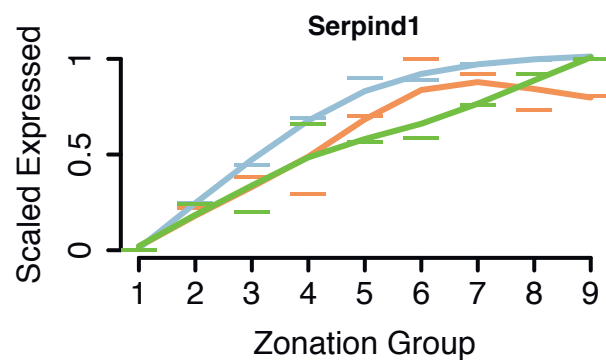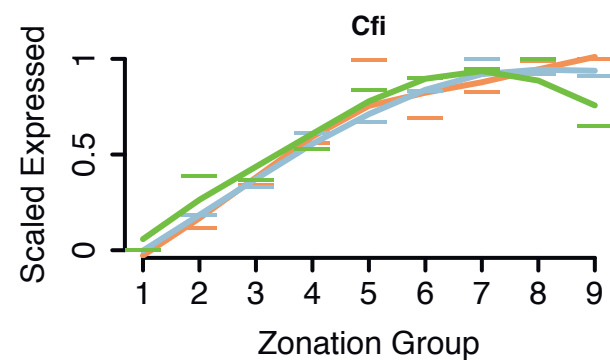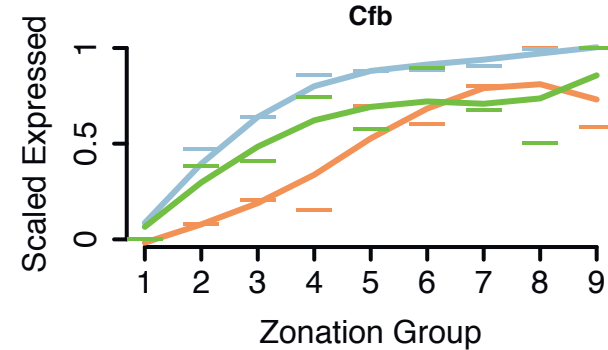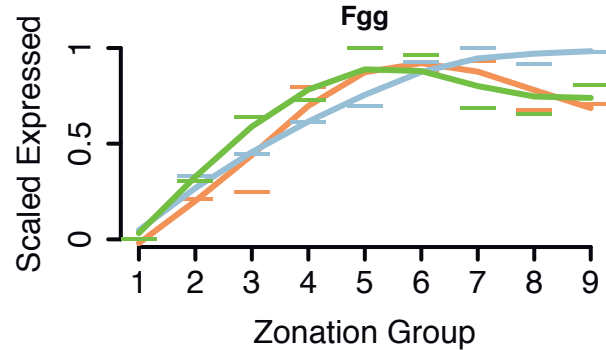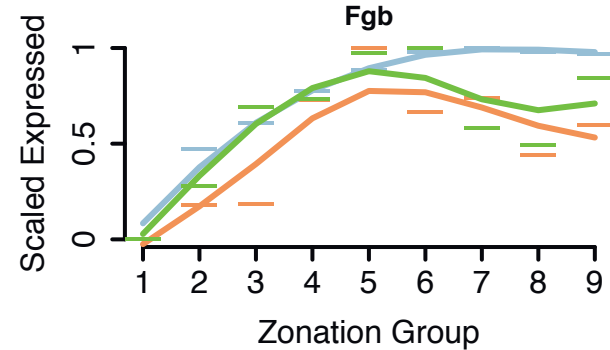**B**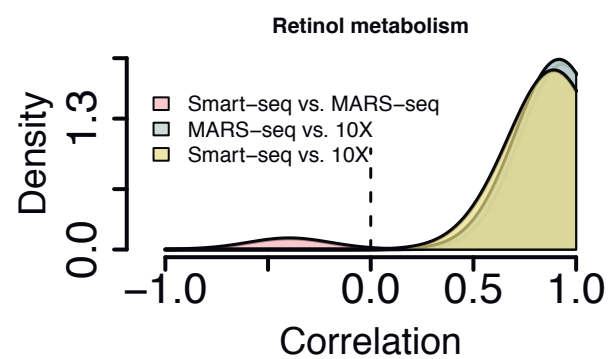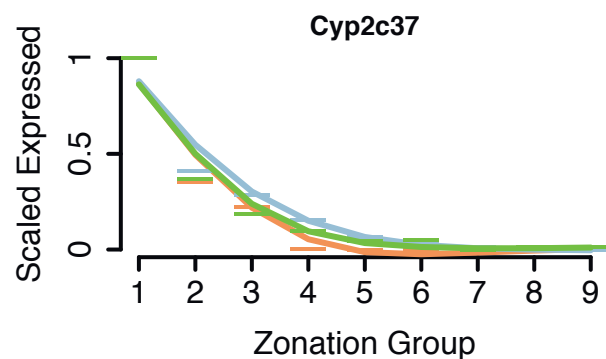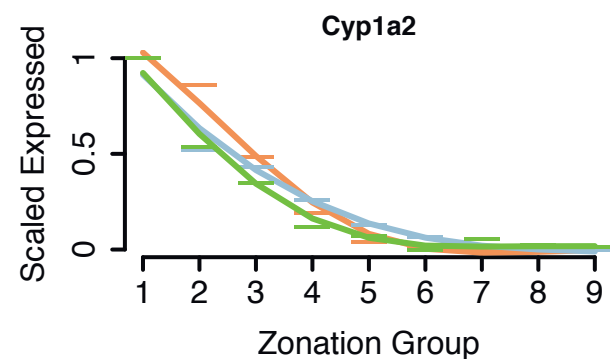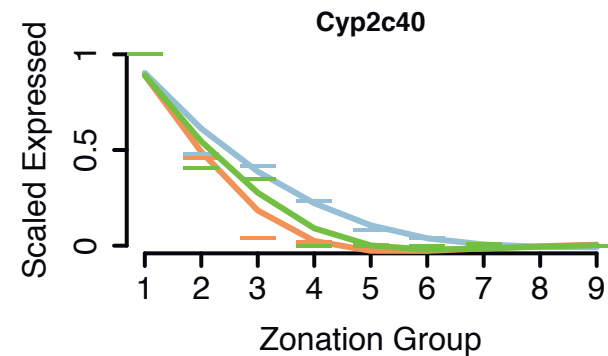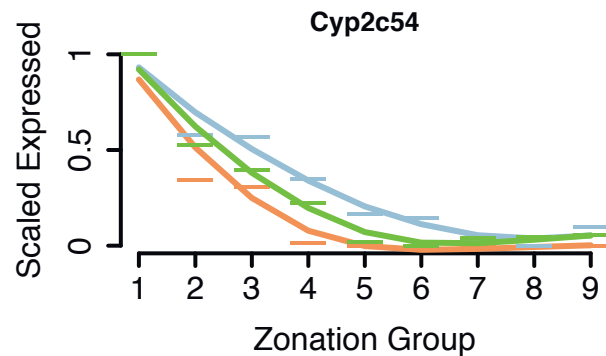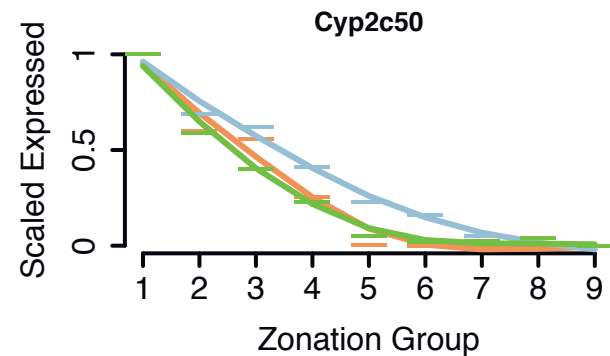**C**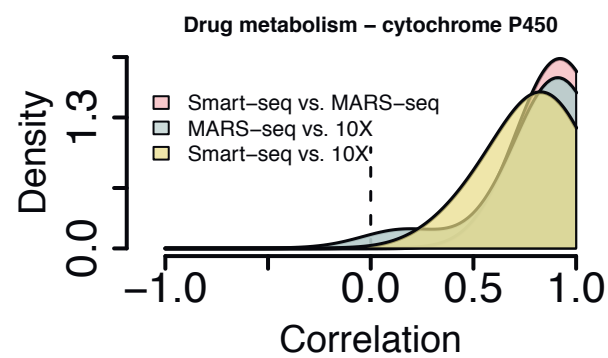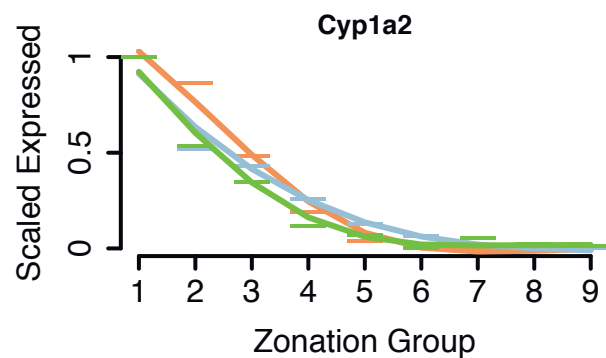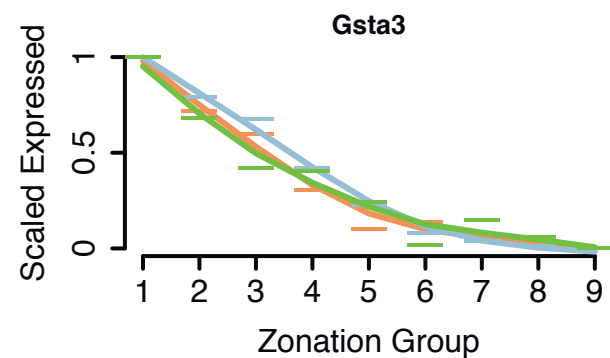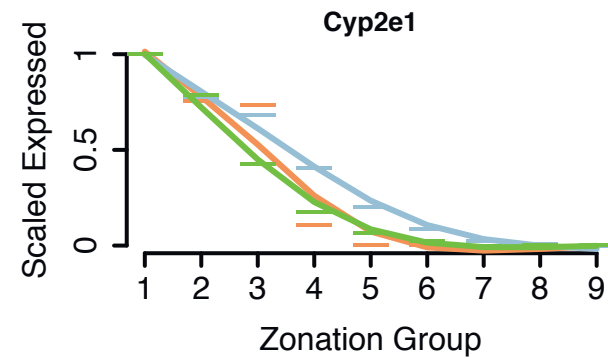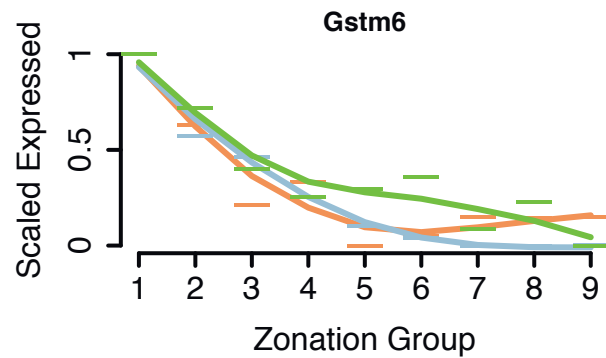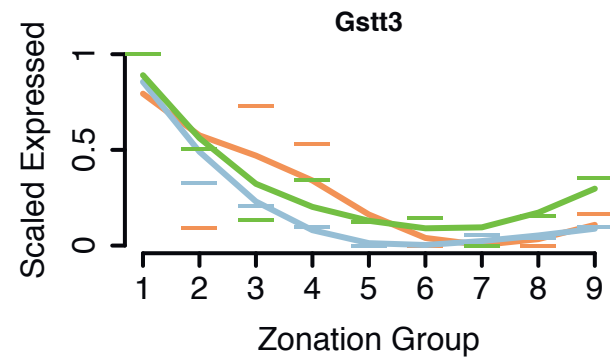

Supplement: S5 Fig — A) Top left: Correlation analysis for genes in the KEGG pathway “Complement and coagulation cascade”. The pairwise correlations are shown for each dataset comparison. Following are plots for the five highest correlated genes in that pathway. B) Similar to (A) but for the “Retinol metabolism” pathway. C) Similar to (A) but for the “Drug metabolism–cytochrome P450” pathway. (PDF) [file pone.0239711.s005.pdf]

**Ugt1a1**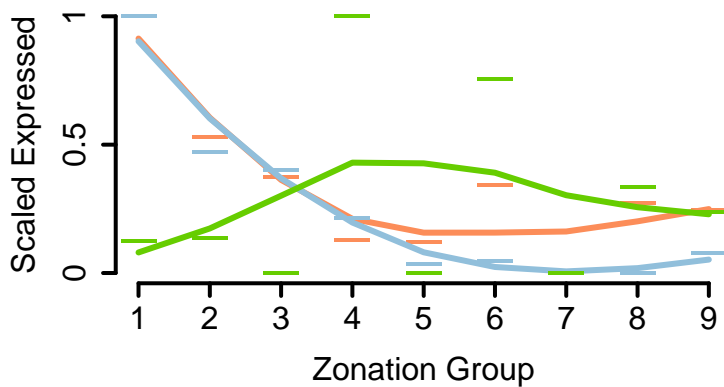**Ugt1a10**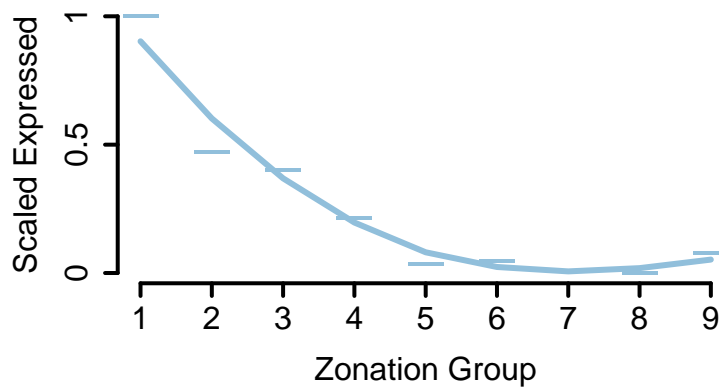**Ugt1a2**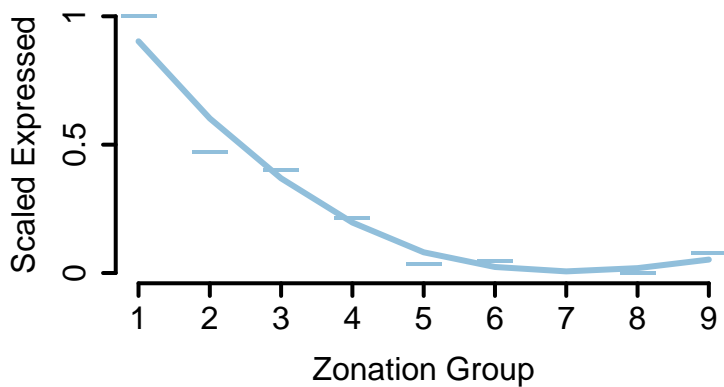**Ugt1a5**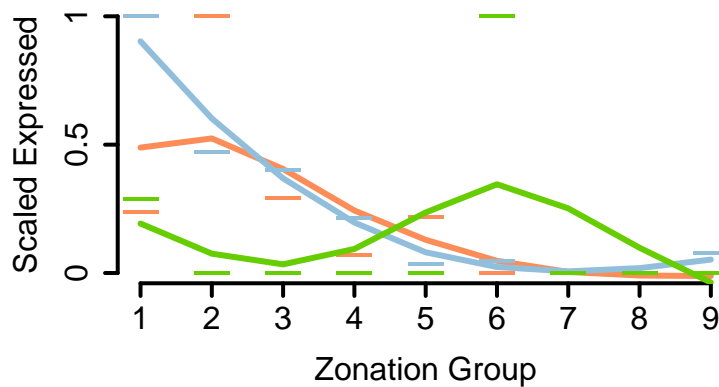**Ugt1a6a**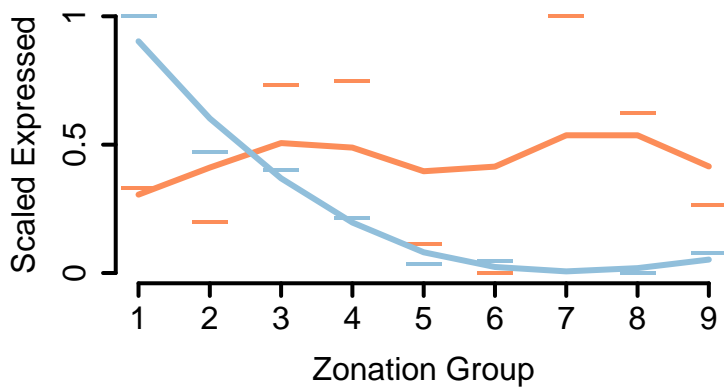**Ugt1a6b**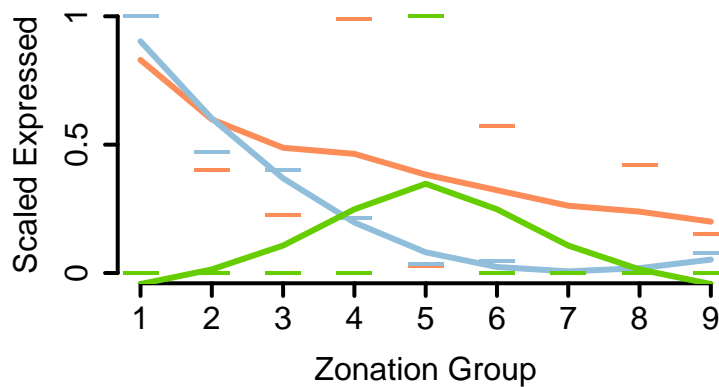**Ugt1a7c**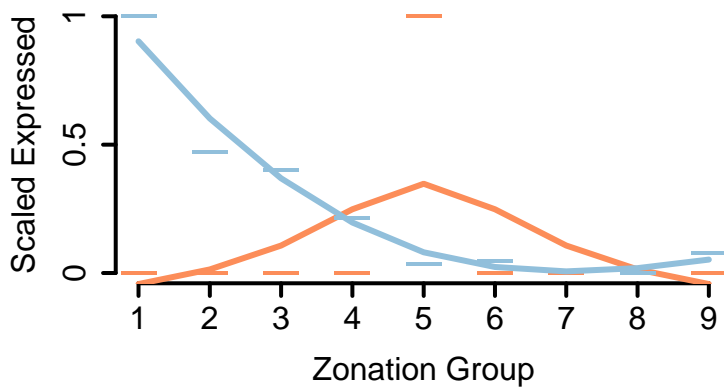**Ugt1a9**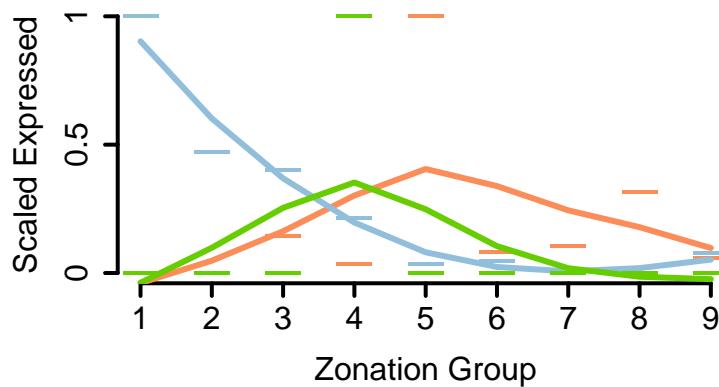

Supplement: S6 Fig — Eight Ugt1a genes that were concatenated in the MARS-seq dataset (blue on all graphs), but can be resolved in the Smart-seq dataset (orange line). (PDF) [file pone.0239711.s006.pdf]
