# Supplementary material for: Reproducibility across single-cell RNA-seq protocols for spatial ordering analysis
Source: PLoS One. 2020 Sep 28;15(9):e0239711. doi: 10.1371/journal.pone.0239711 (PMC7521718; doi:10.1371/journal.pone.0239711)
Supplement: S1 File — (PDF) [file pone.0239711.s009.pdf]

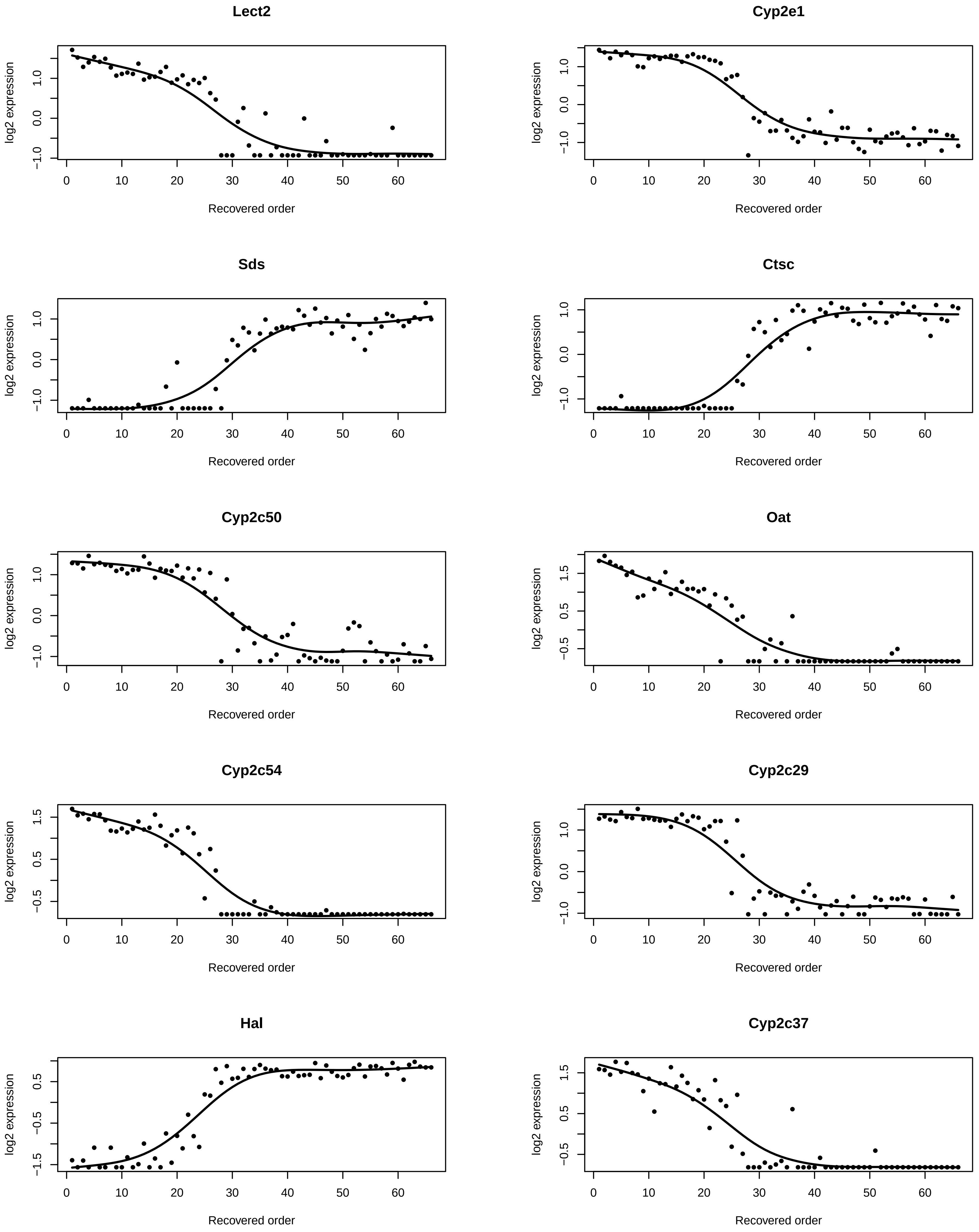
Lect2
Cyp2e1
Sds
Ctsc
Cyp2c50
Oat
Cyp2c54
Cyp2c29
Hal
Cyp2c37
log2 expression
Recovered order
0
10
20
30
40
50
60
−1.0
0.0
1.0
−0.5
0.5
1.5

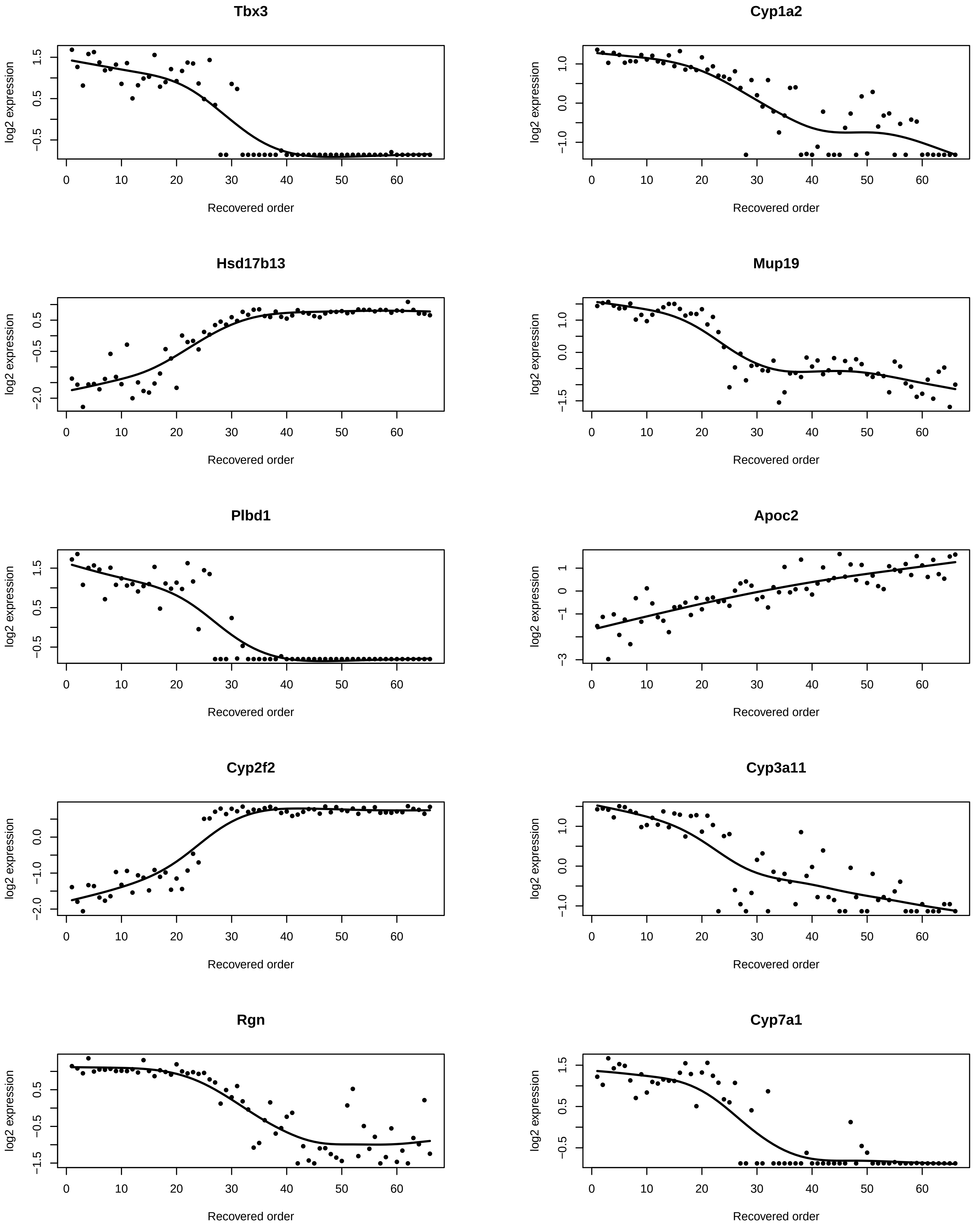
Tbx3
log2 expression
-0.5 0.5 1.5
Recovered order
0 10 20 30 40 50 60
Cyp1a2
log2 expression
-1.0 0.0 1.0
Recovered order
0 10 20 30 40 50 60
Hsd17b13
log2 expression
-2.0 -0.5 0.5
Recovered order
0 10 20 30 40 50 60
Mup19
log2 expression
-1.5 0.0 1.0
Recovered order
0 10 20 30 40 50 60
Plbd1
log2 expression
-0.5 0.5 1.5
Recovered order
0 10 20 30 40 50 60
Apoc2
log2 expression
-3 -1 0 1
Recovered order
0 10 20 30 40 50 60
Cyp2f2
log2 expression
-2.0 -1.0 0.0
Recovered order
0 10 20 30 40 50 60
Cyp3a11
log2 expression
-1.0 0.0 1.0
Recovered order
0 10 20 30 40 50 60
Rgn
log2 expression
-1.5 -0.5 0.5
Recovered order
0 10 20 30 40 50 60
Cyp7a1
log2 expression
-0.5 0.5 1.5
Recovered order
0 10 20 30 40 50 60

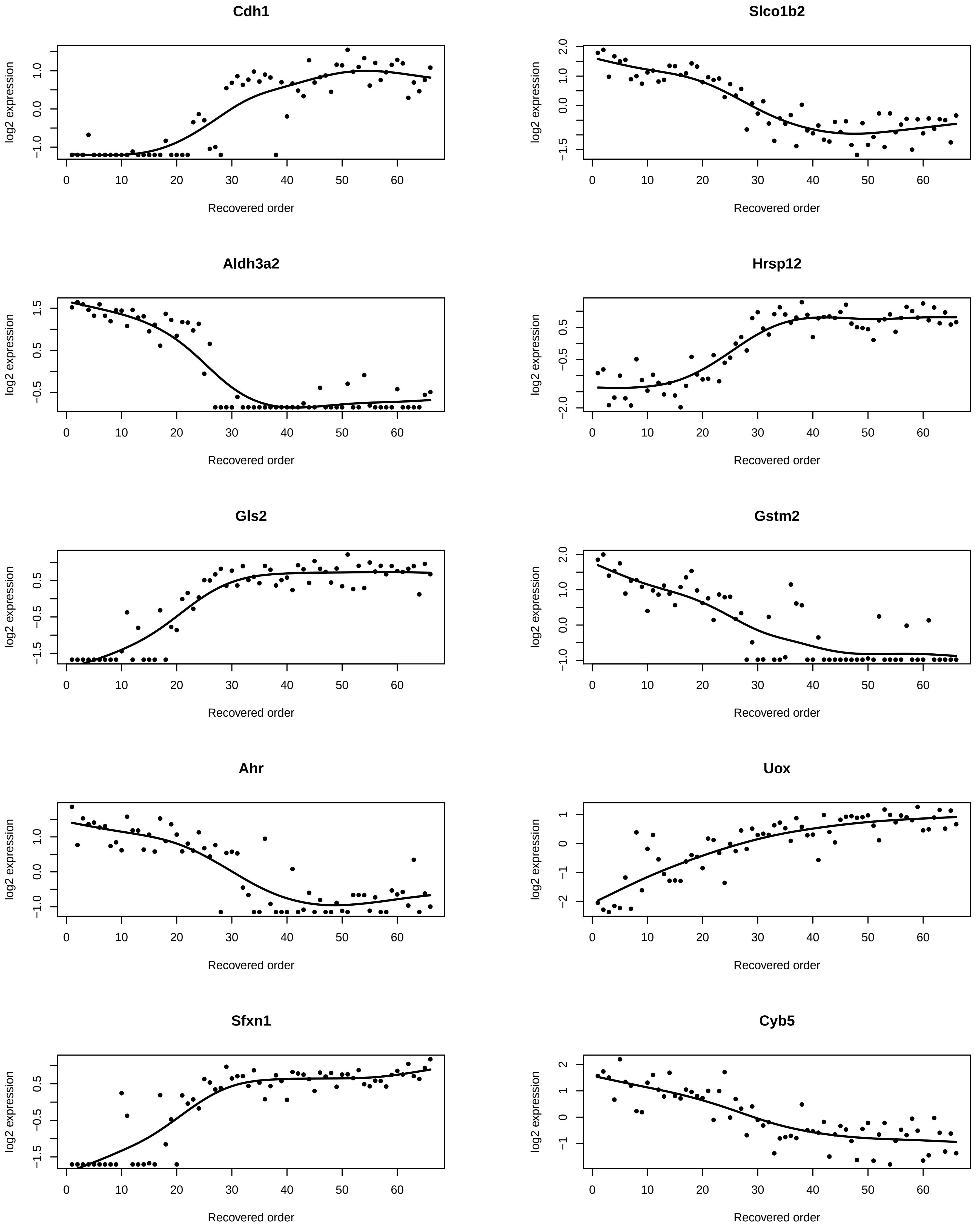
Cdh1
Slco1b2
Aldh3a2
Hrsp12
Gls2
Gstm2
Ahr
Uox
Sfxn1
Cyb5
log2 expression
Recovered order

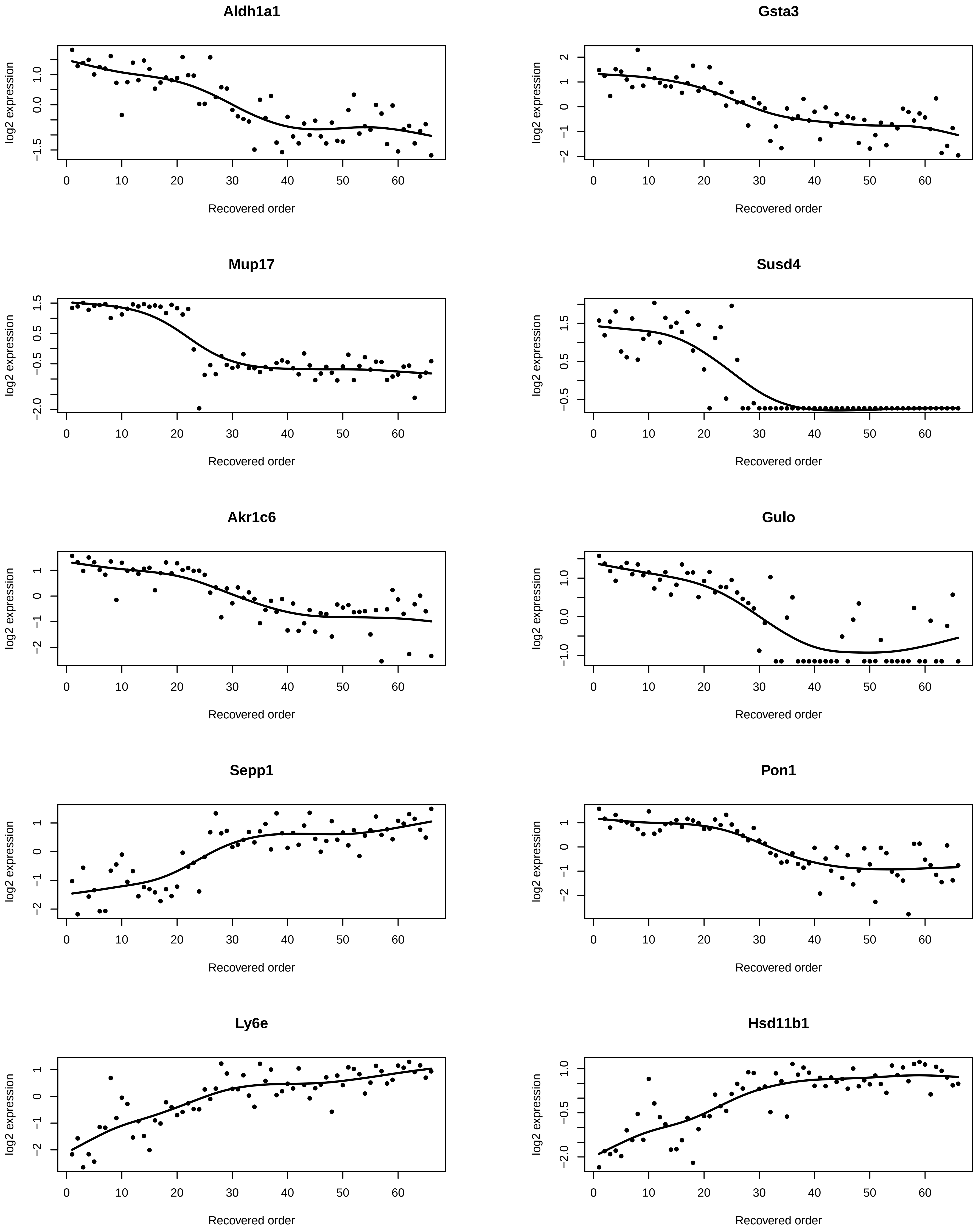
Aldh1a1
Gsta3
Mup17
Susd4
Akr1c6
Gulo
Sepp1
Pon1
Ly6e
Hsd11b1
log2 expression
Recovered order

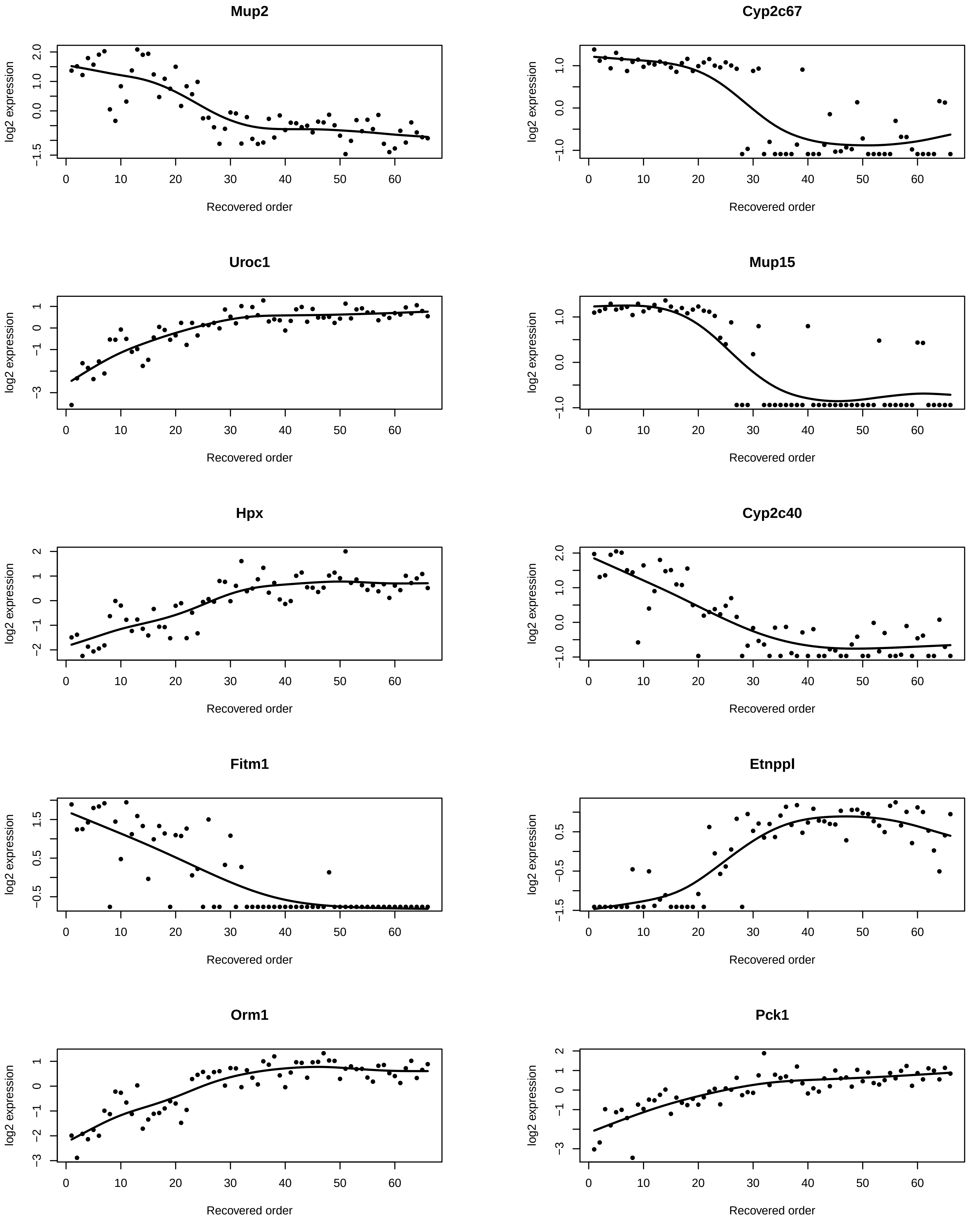

Mup2
Cyp2c67
Uroc1
Mup15
Hpx
Cyp2c40
Fitm1
Etnppl
Orm1
Pck1
log2 expression
Recovered order

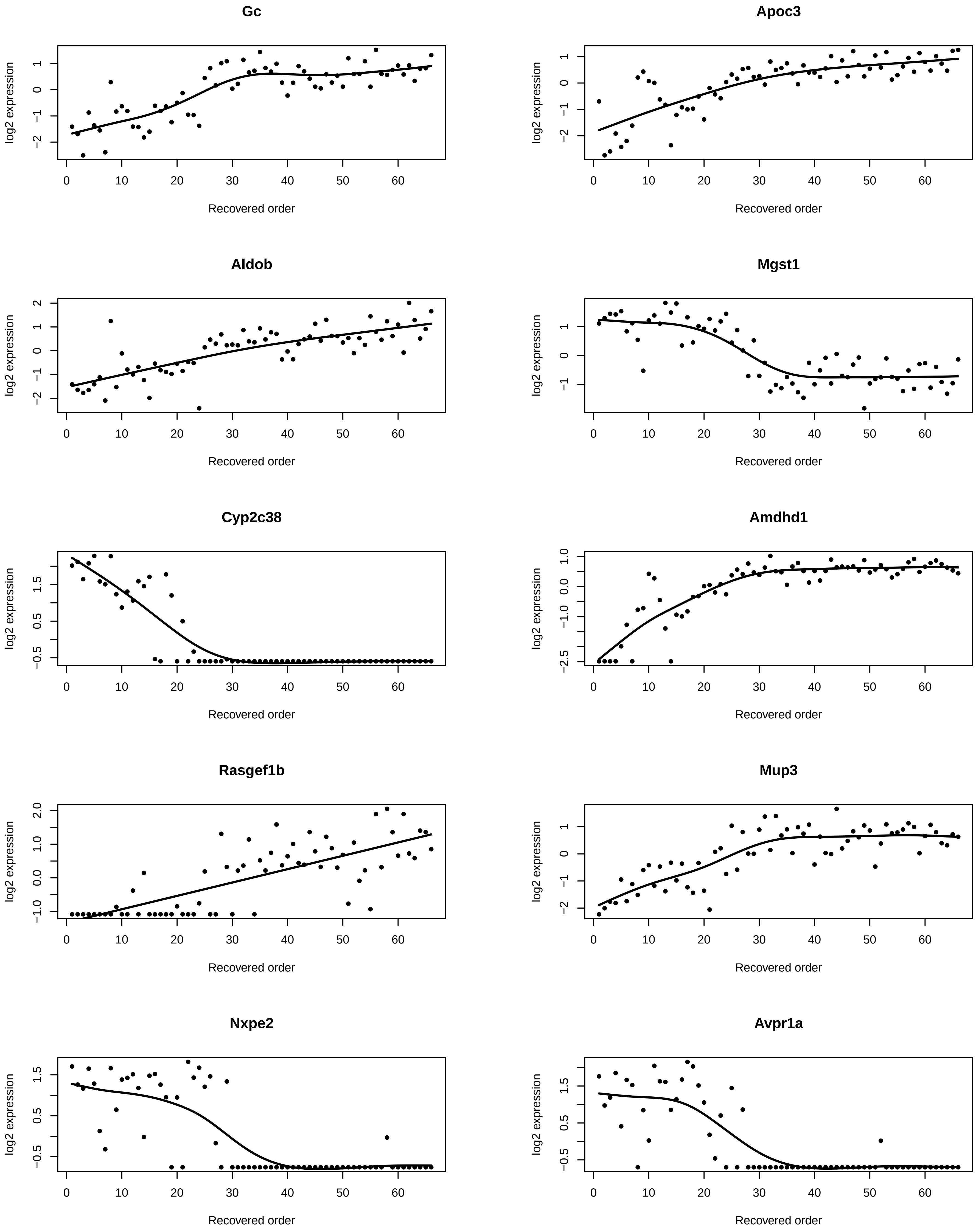
Gc
Apoc3
Aldob
Mgst1
Cyp2c38
Amdhd1
Rasgef1b
Mup3
Nxpe2
Avpr1a
log2 expression
Recovered order

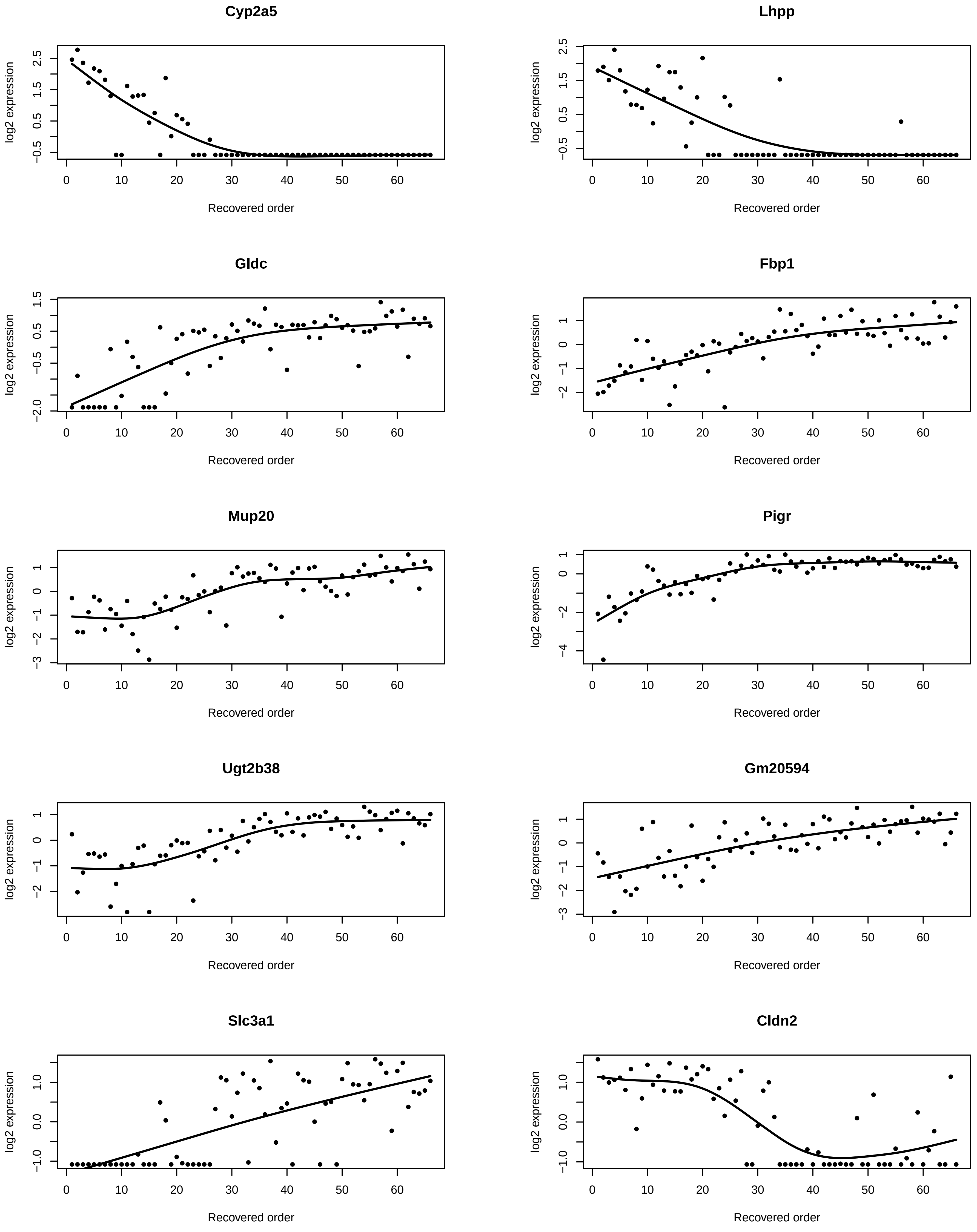

Cyp2a5
Lhpp
Gldc
Fbp1
Mup20
Pigr
Ugt2b38
Gm20594
Slc3a1
Cldn2
log2 expression
Recovered order

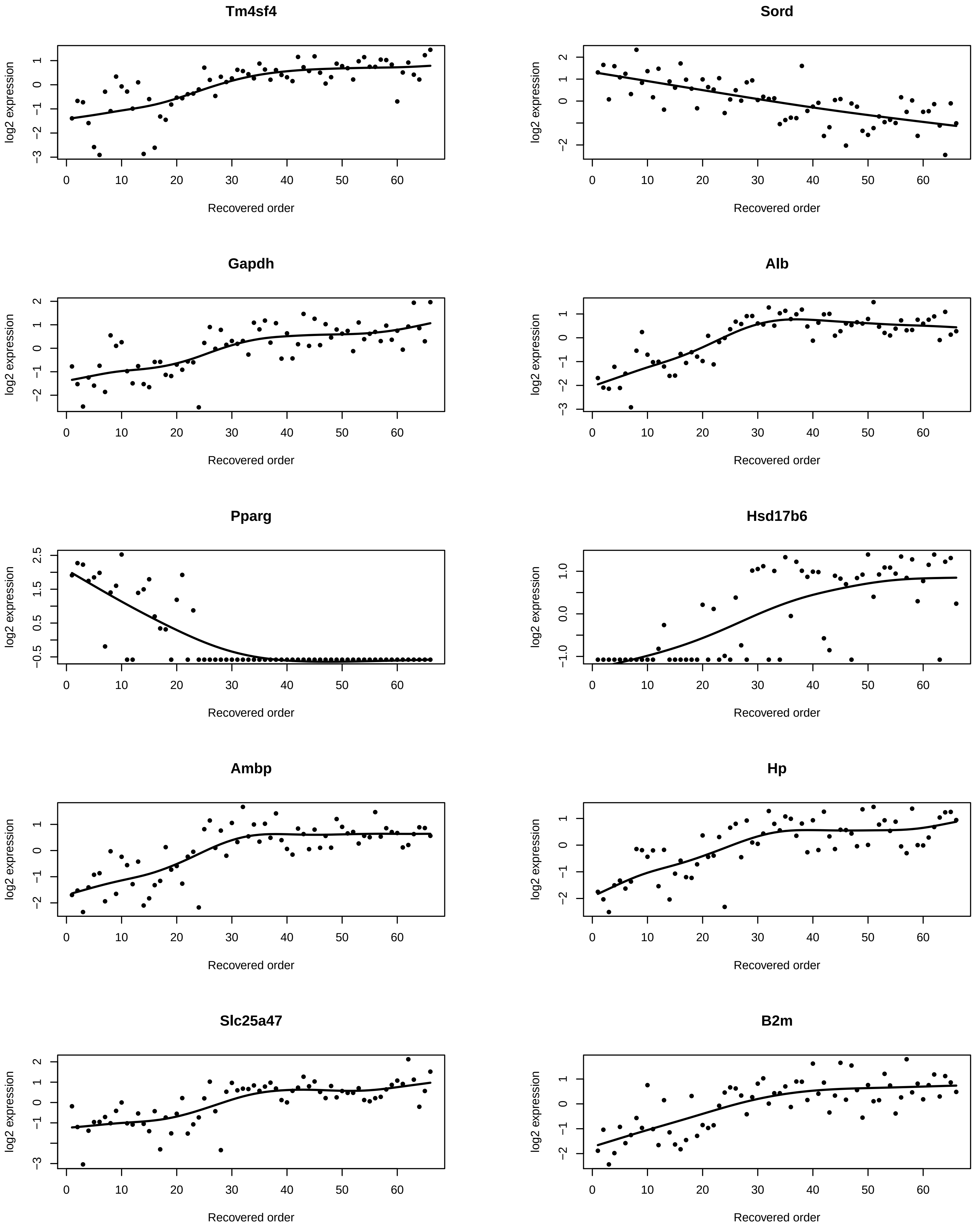
Tm4sf4
Sord
Gapdh
Alb
Pparg
Hsd17b6
Ambp
Hp
Slc25a47
B2m
log2 expression
Recovered order

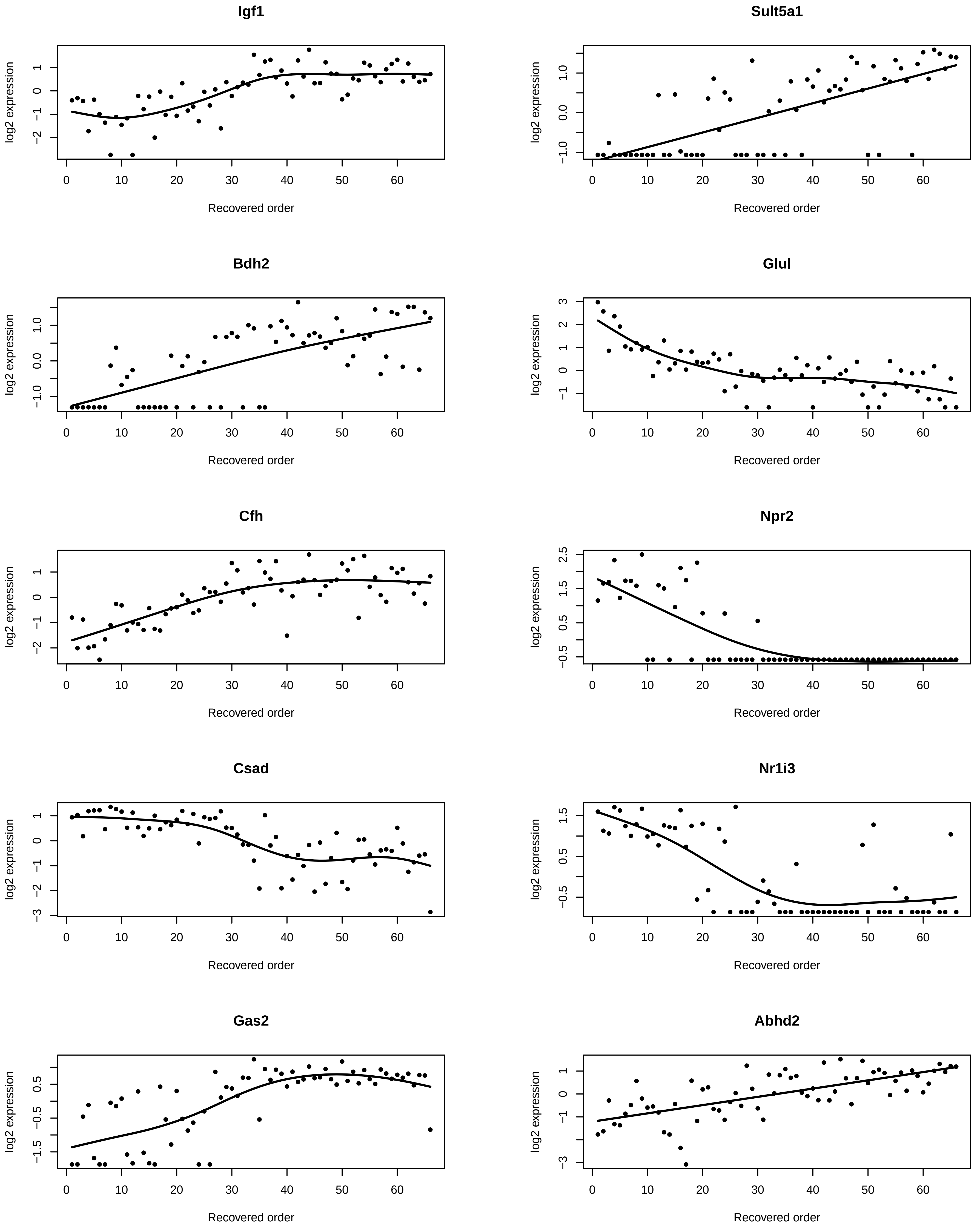
Igf1
Sult5a1
Bdh2
Glul
Cfh
Npr2
Csad
Nr1i3
Gas2
Abhd2
log2 expression
Recovered order

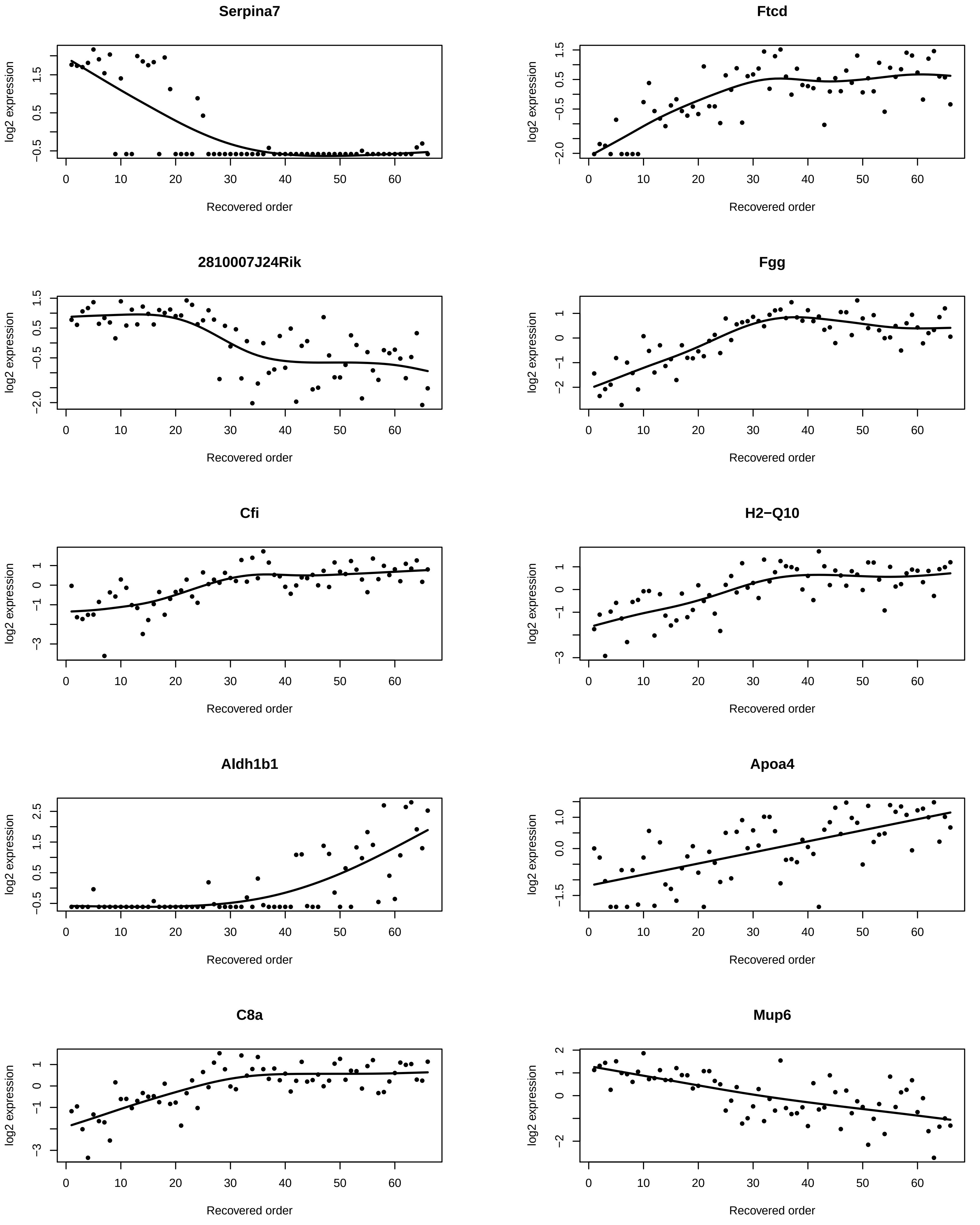

Serpina7
Ftcd
2810007J24Rik
Fgg
Cfi
H2−Q10
Aldh1b1
Apoa4
C8a
Mup6
log2 expression
Recovered order

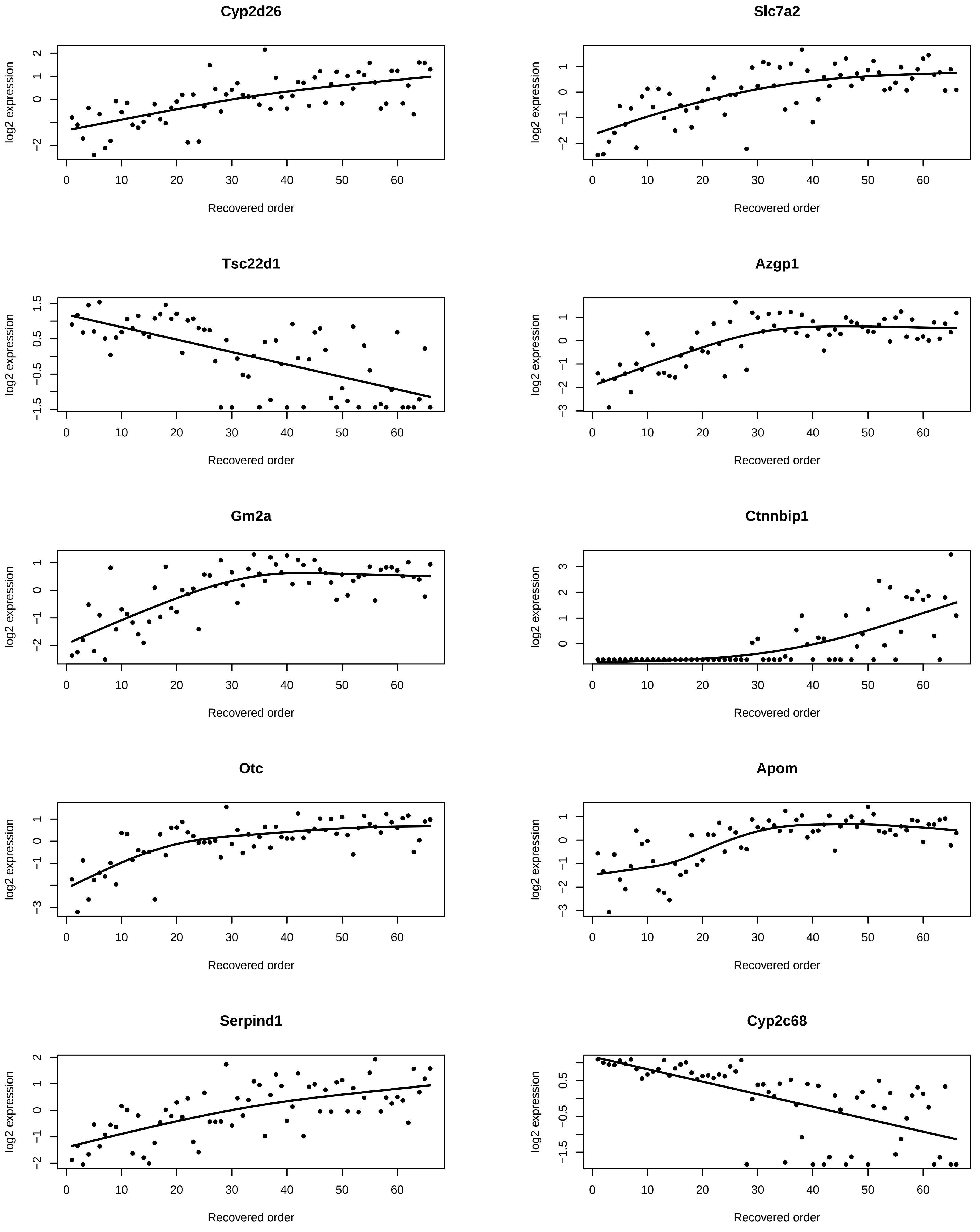
Cyp2d26
Slc7a2
Tsc22d1
Azgp1
Gm2a
Ctnnbip1
Otc
Apom
Serpind1
Cyp2c68
log2 expression
Recovered order

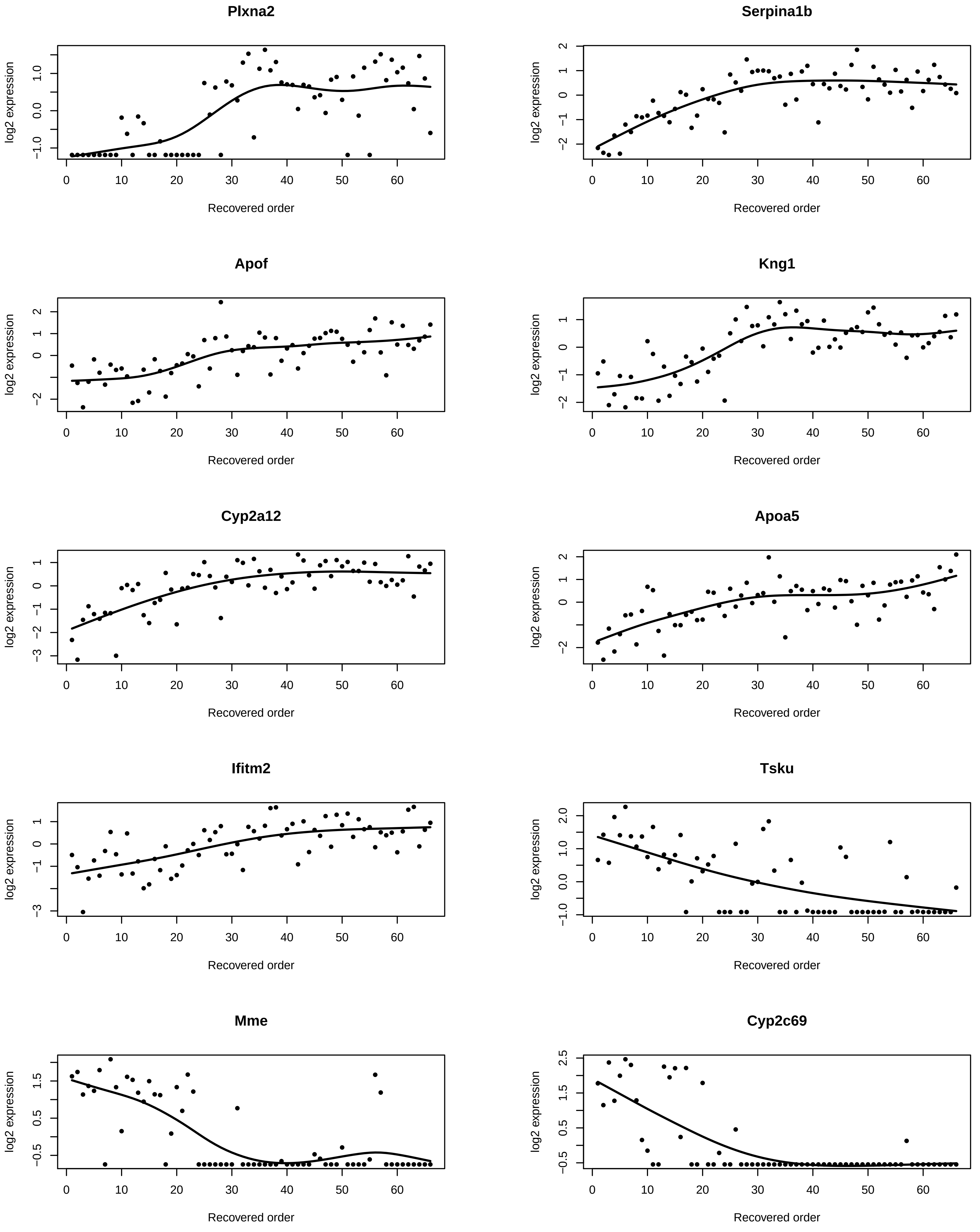
Plxna2
Serpina1b
Apof
Kng1
Cyp2a12
Apoa5
Ifitm2
Tsku
Mme
Cyp2c69
log2 expression
Recovered order

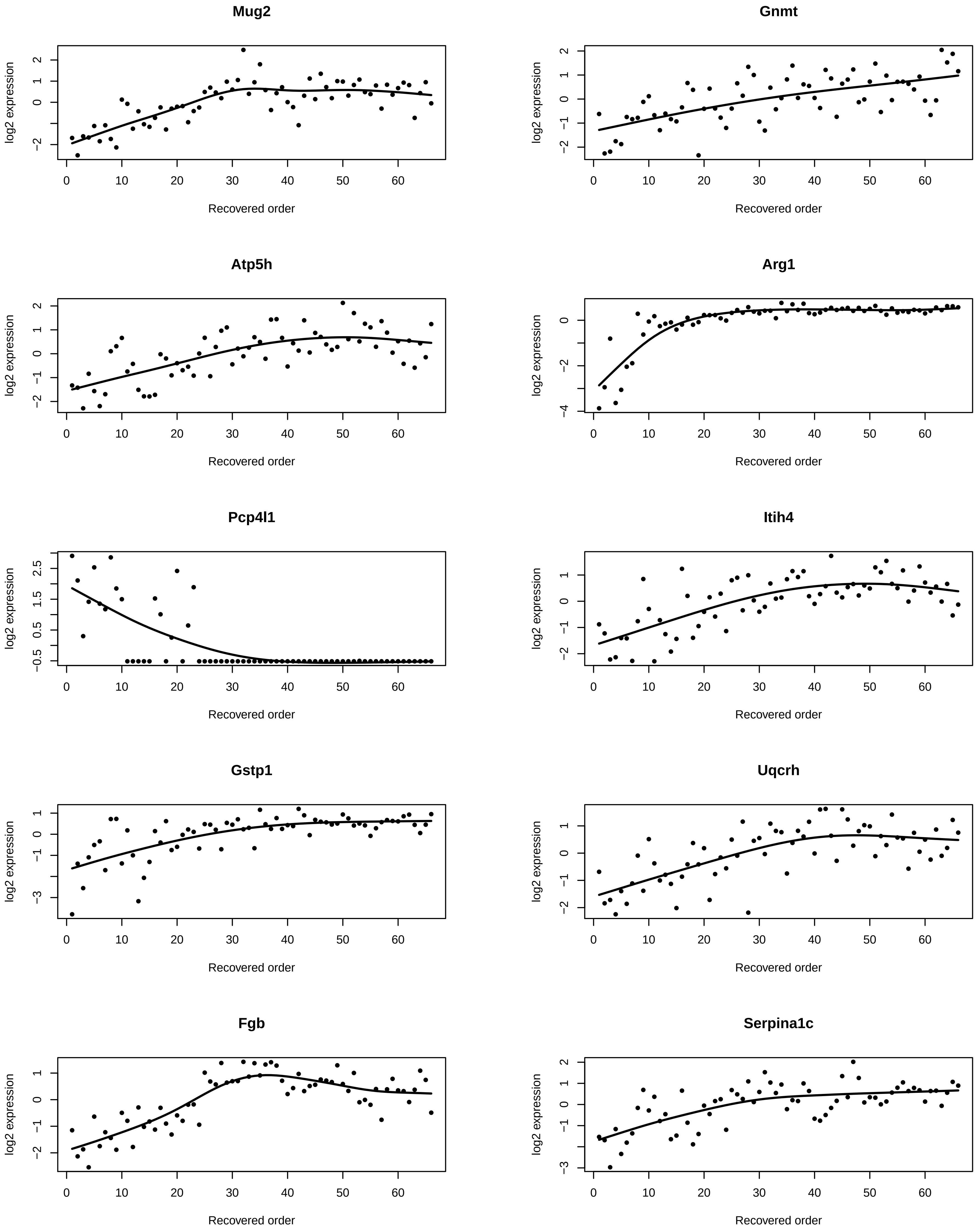
Mug2
log2 expression
Recovered order
Gnmt
log2 expression
Recovered order
Atp5h
log2 expression
Recovered order
Arg1
log2 expression
Recovered order
Pcp4l1
log2 expression
Recovered order
Itih4
log2 expression
Recovered order
Gstp1
log2 expression
Recovered order
Uqcrh
log2 expression
Recovered order
Fgb
log2 expression
Recovered order
Serpina1c
log2 expression
Recovered order

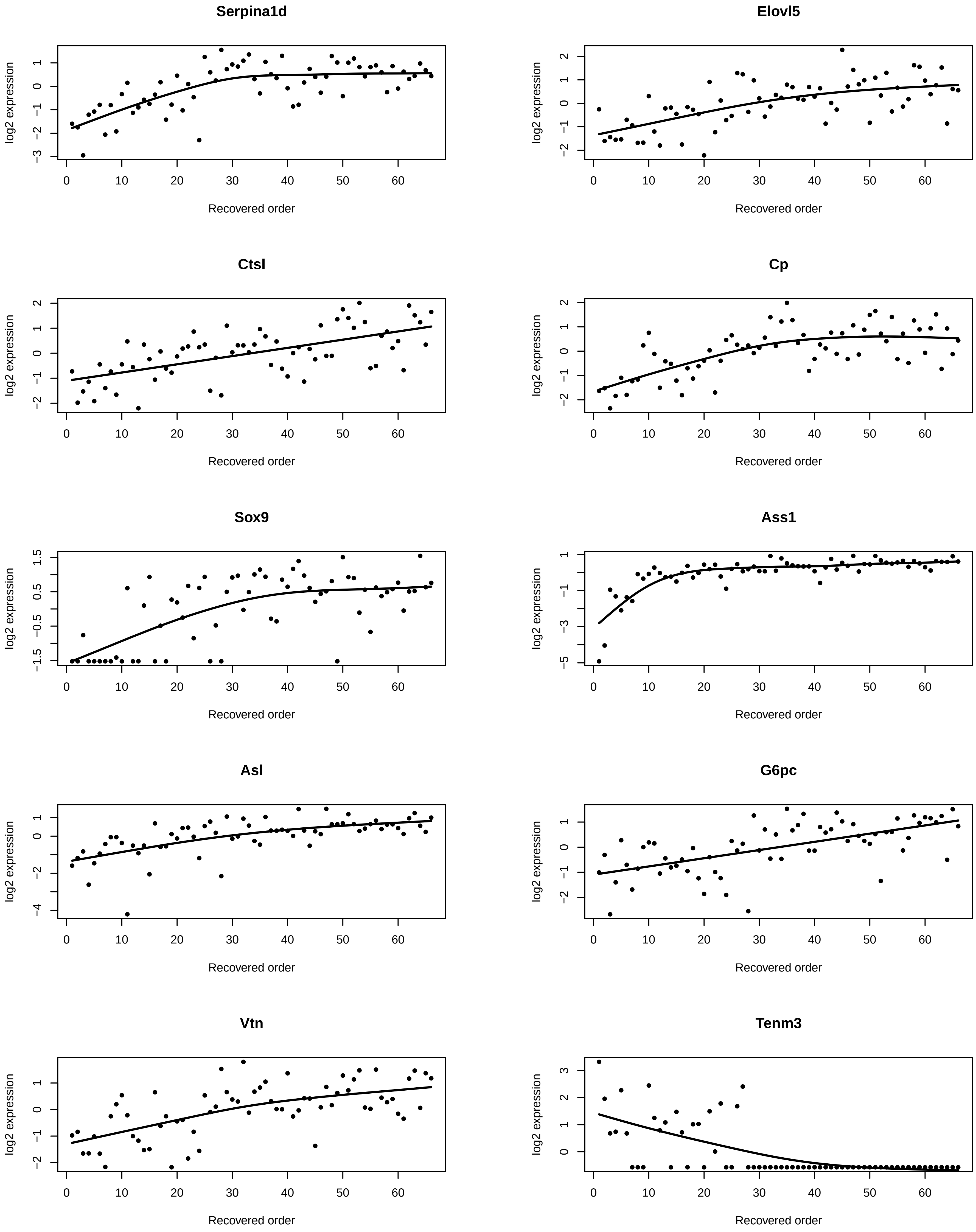
Serpina1d
log2 expression
Recovered order
Elovl5
log2 expression
Recovered order
Ctsl
log2 expression
Recovered order
Cp
log2 expression
Recovered order
Sox9
log2 expression
Recovered order
Ass1
log2 expression
Recovered order
Asl
log2 expression
Recovered order
G6pc
log2 expression
Recovered order
Vtn
log2 expression
Recovered order
Tenm3
log2 expression
Recovered order

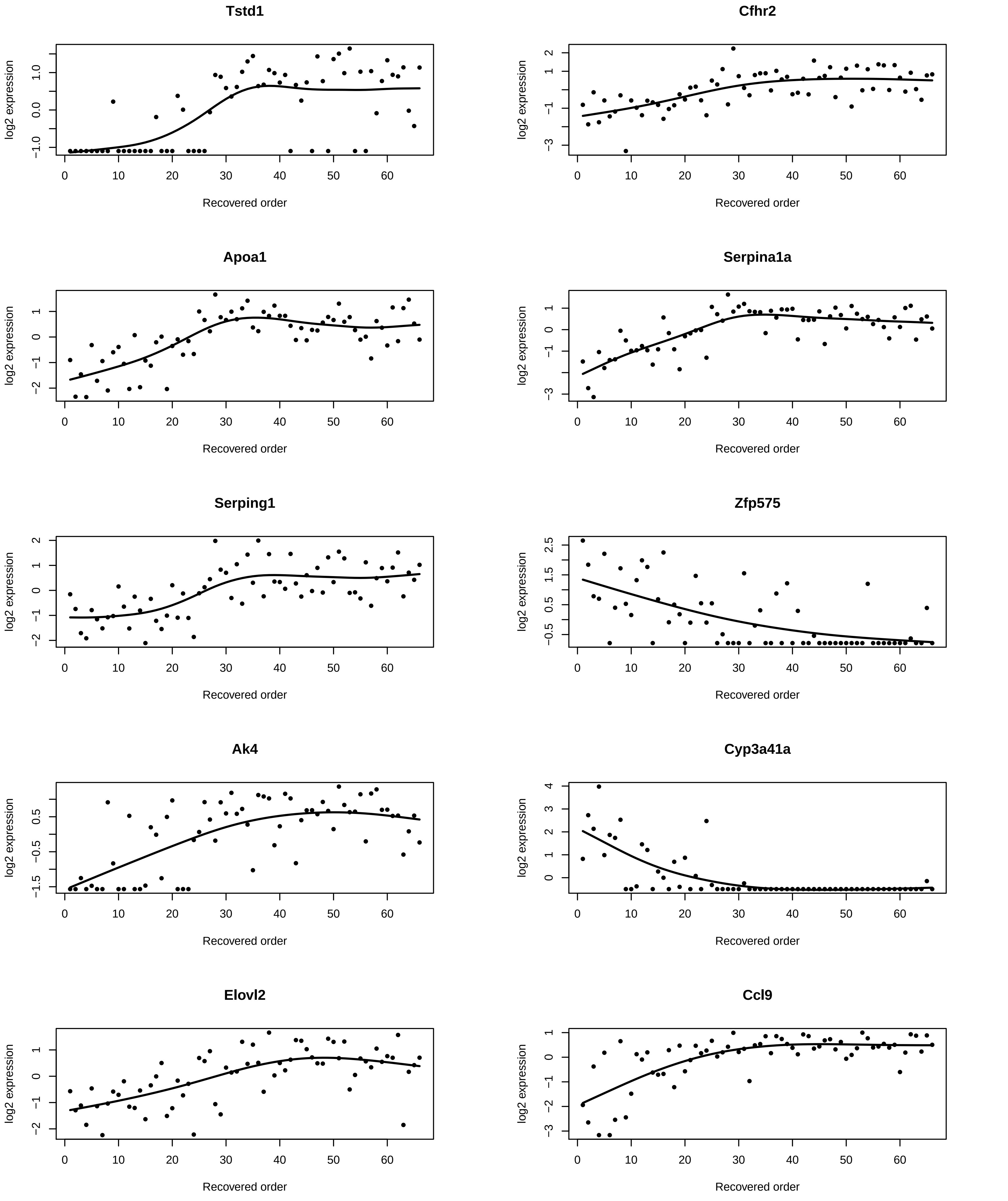
Tstd1
Cfhr2
Apoa1
Serpina1a
Serping1
Zfp575
Ak4
Cyp3a41a
Elovl2
Ccl9
log2 expression
Recovered order

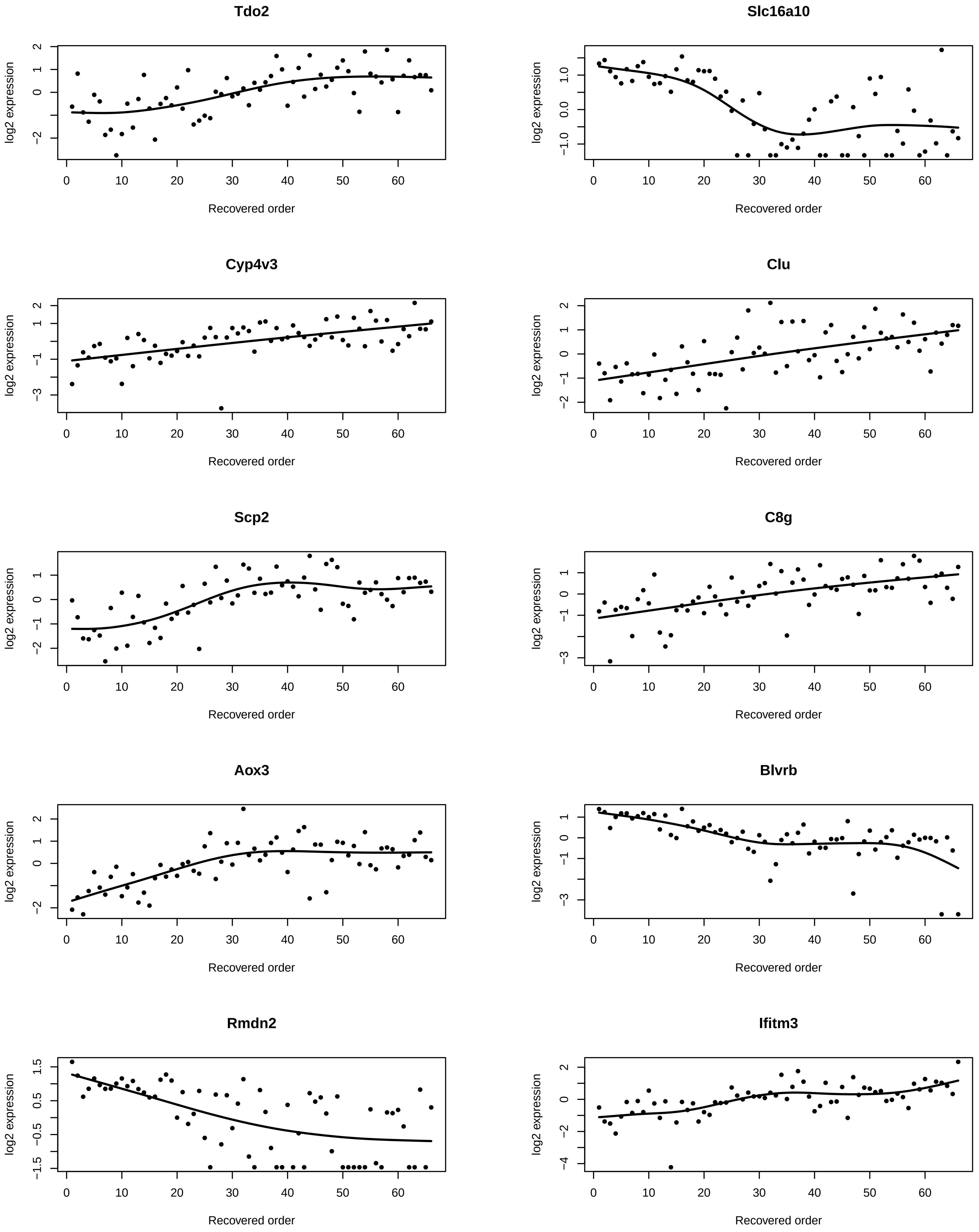

Tdo2
Slc16a10
Cyp4v3
Clu
Scp2
C8g
Aox3
Blvrb
Rmdn2
Ifitm3
log2 expression
Recovered order

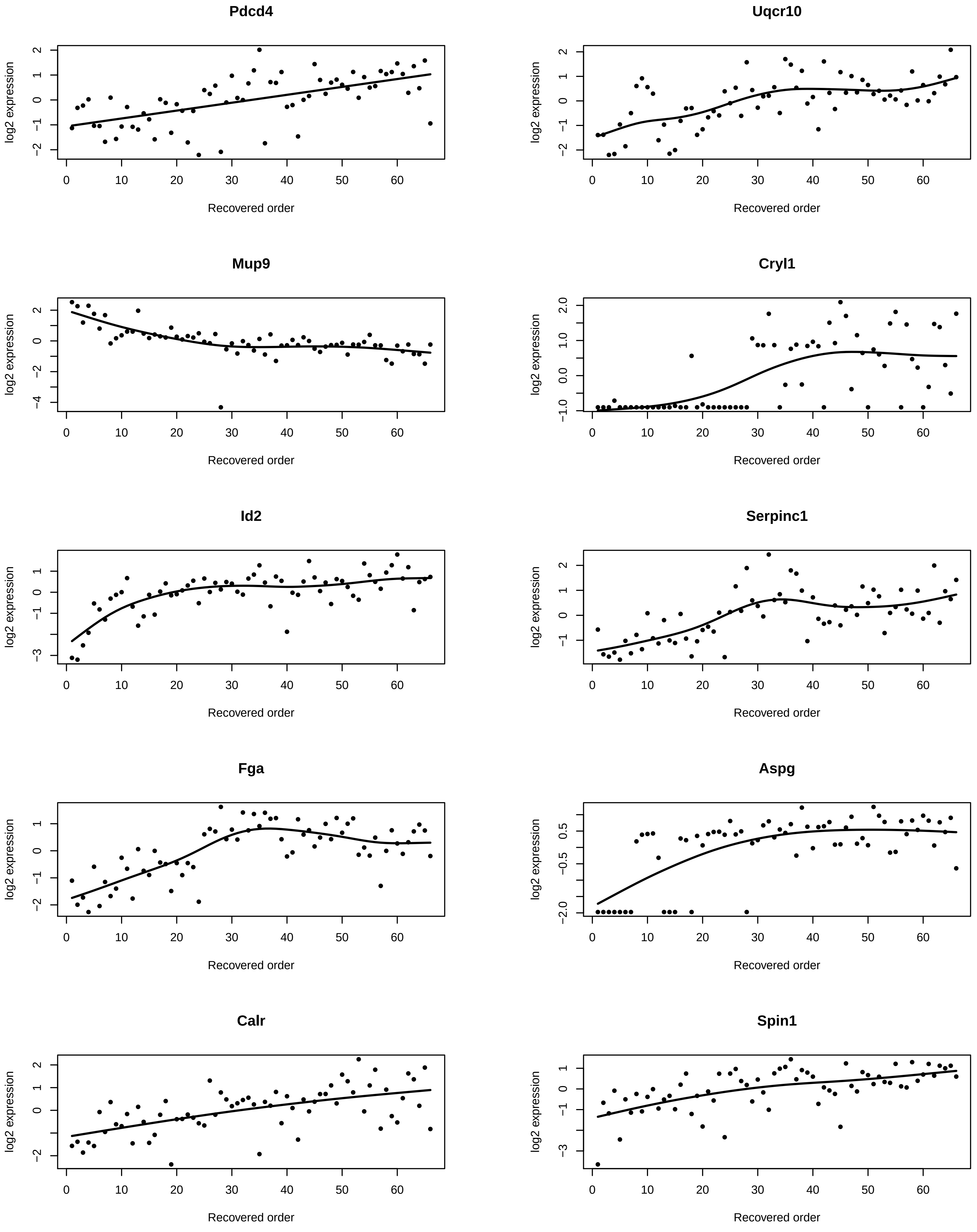
Pdcd4
Uqcr10
Mup9
Cryl1
Id2
Serpinc1
Fga
Aspg
Calr
Spin1
log2 expression
Recovered order

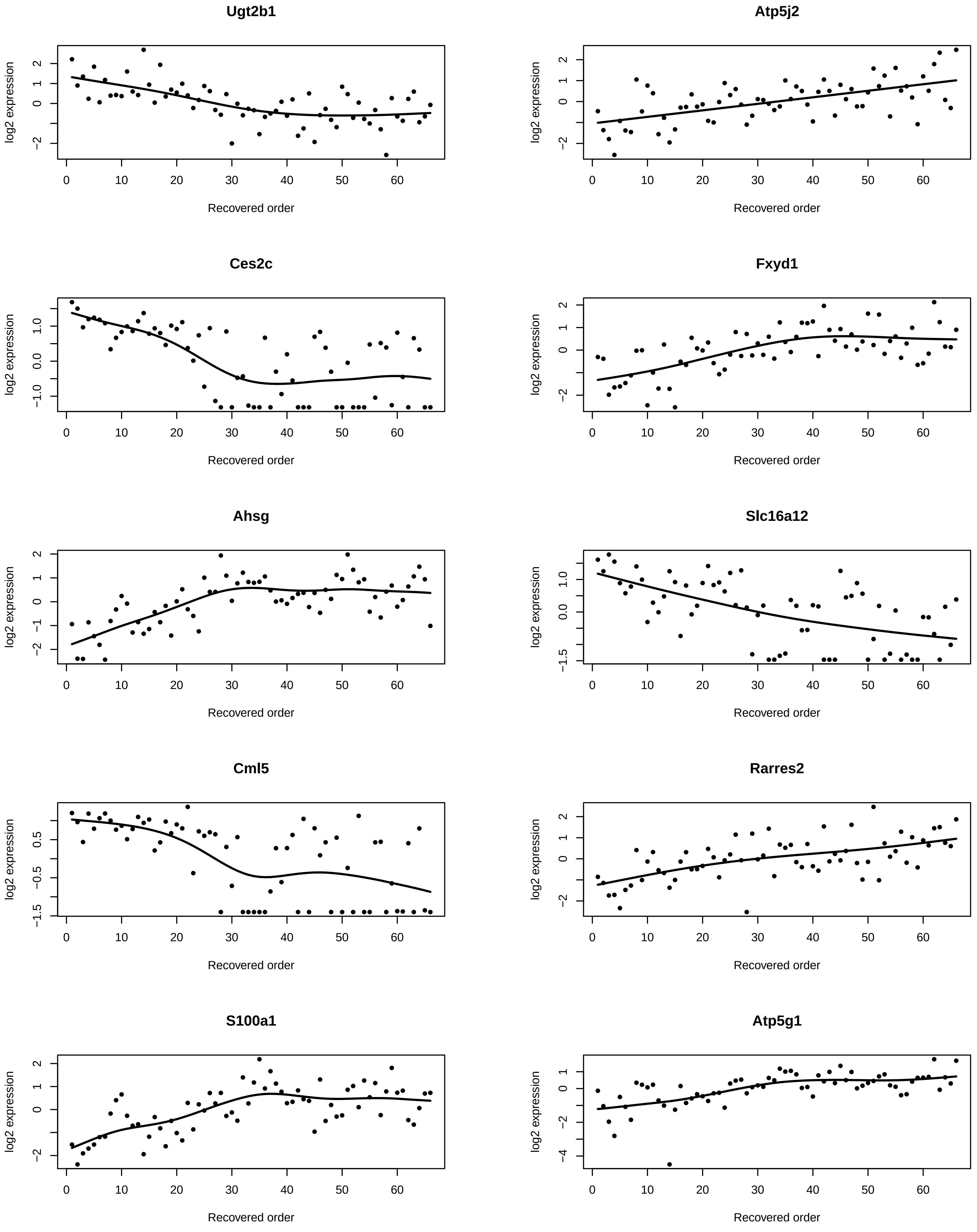
Ugt2b1
Atp5j2
Ces2c
Fxyd1
Ahsg
Slc16a12
Cml5
Rarres2
S100a1
Atp5g1
log2 expression
Recovered order
0
10
20
30
40
50
60

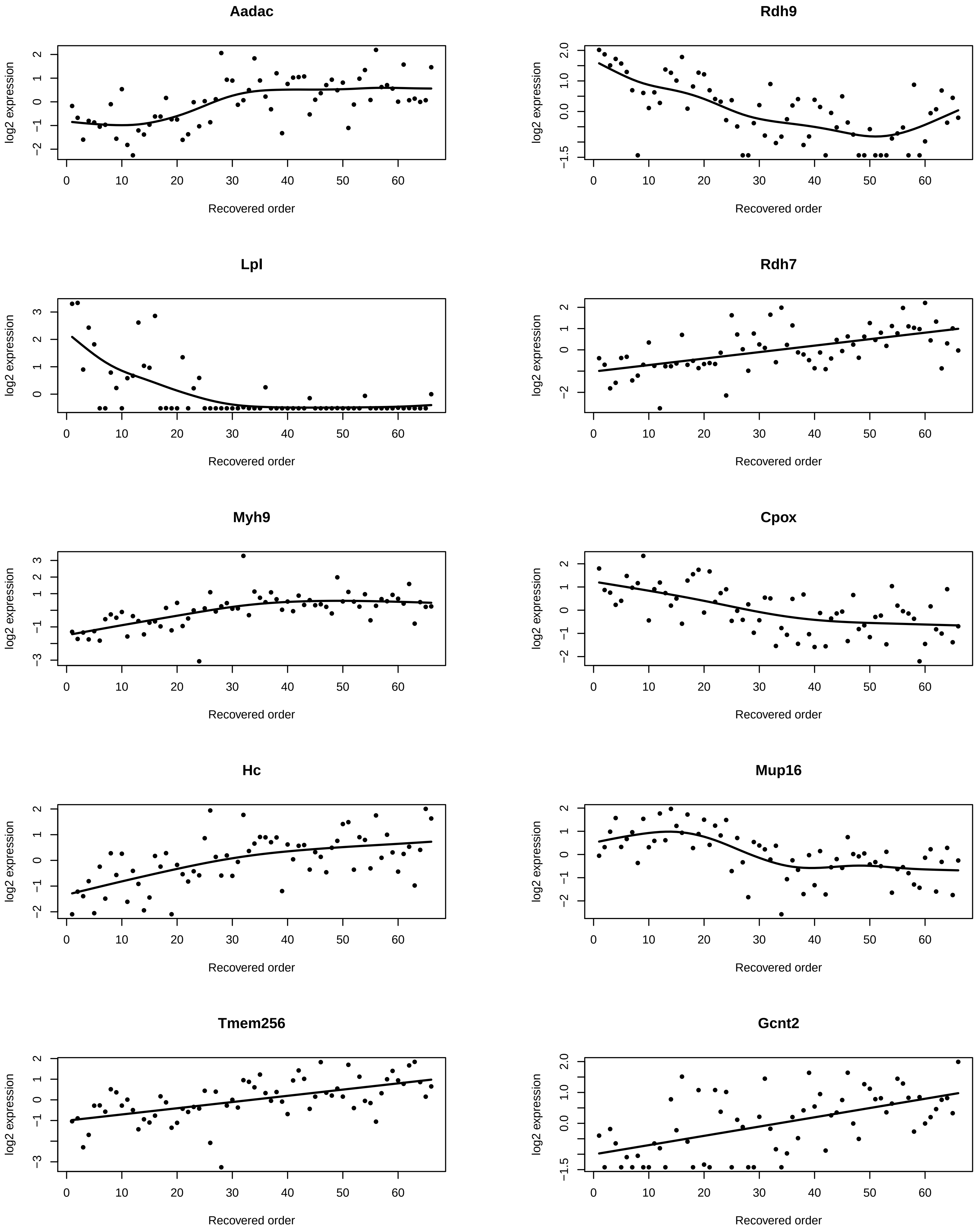
Aadac
Rdh9
Lpl
Rdh7
Myh9
Cpox
Hc
Mup16
Tmem256
Gcnt2
log2 expression
Recovered order
0
10
20
30
40
50
60

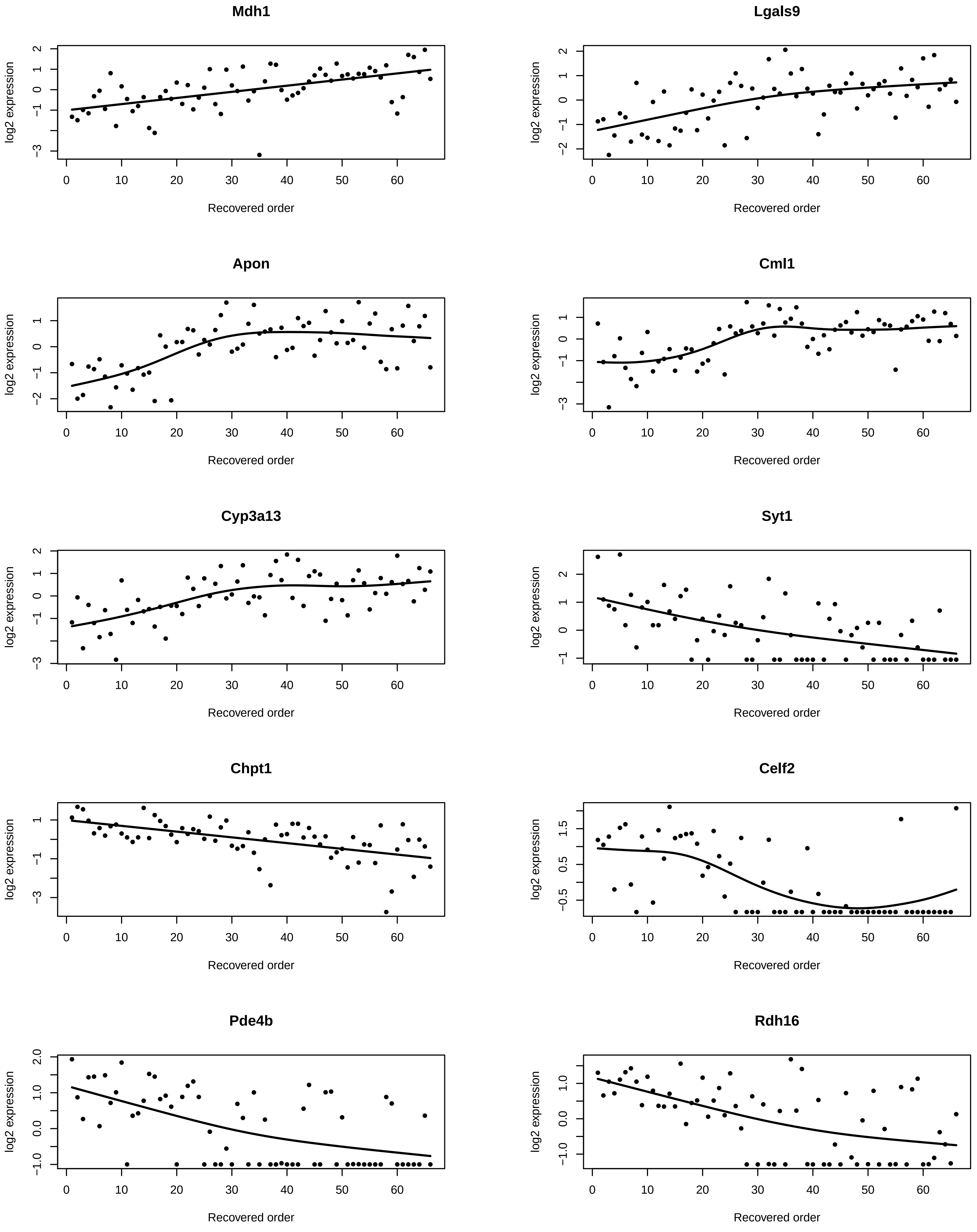
Mdh1
log2 expression
Recovered order
Lgals9
log2 expression
Recovered order
Apon
log2 expression
Recovered order
Cml1
log2 expression
Recovered order
Cyp3a13
log2 expression
Recovered order
Syt1
log2 expression
Recovered order
Chpt1
log2 expression
Recovered order
Celf2
log2 expression
Recovered order
Pde4b
log2 expression
Recovered order
Rdh16
log2 expression
Recovered order

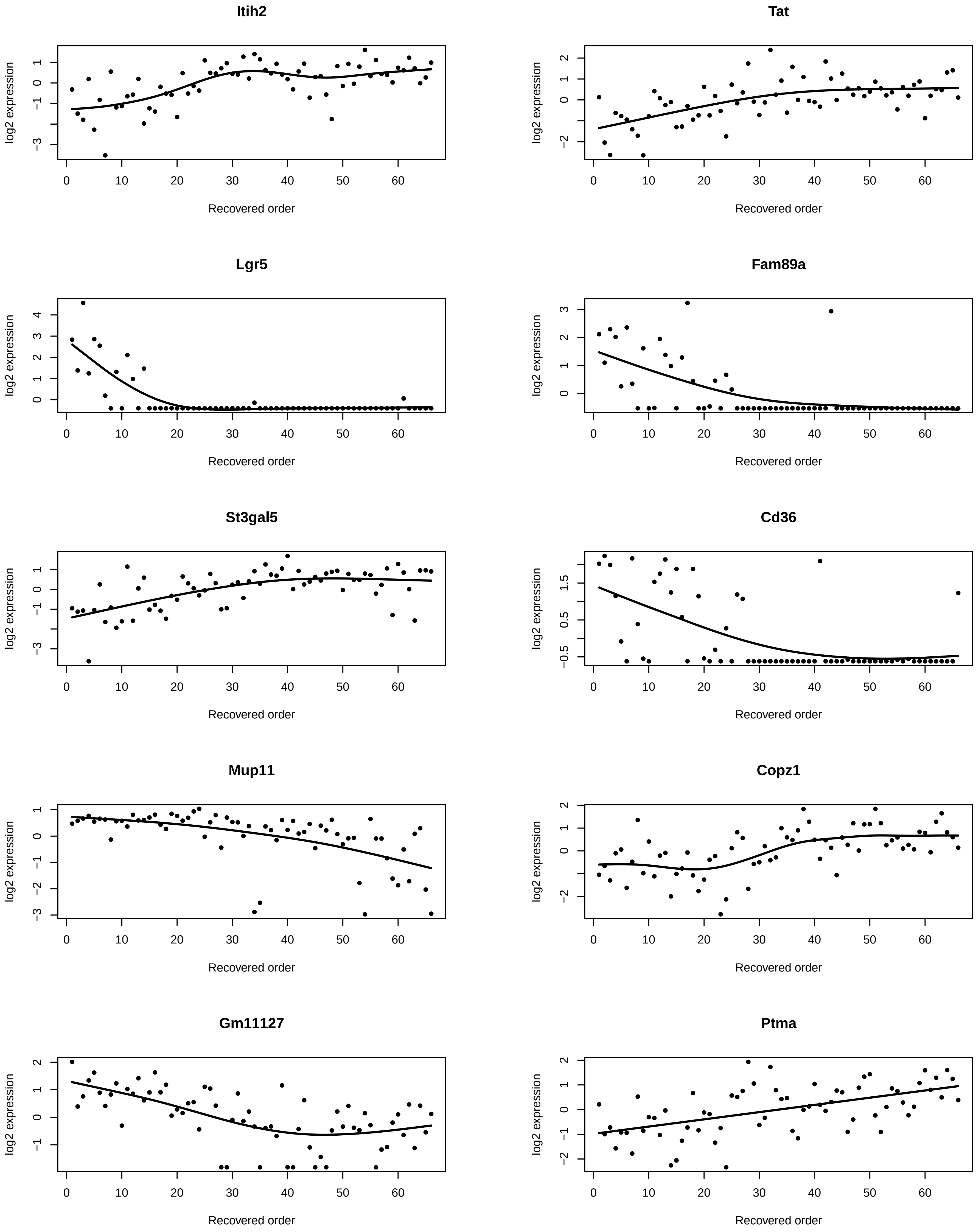
Itih2
Tat
Lgr5
Fam89a
St3gal5
Cd36
Mup11
Copz1
Gm11127
Ptma
log2 expression
Recovered order

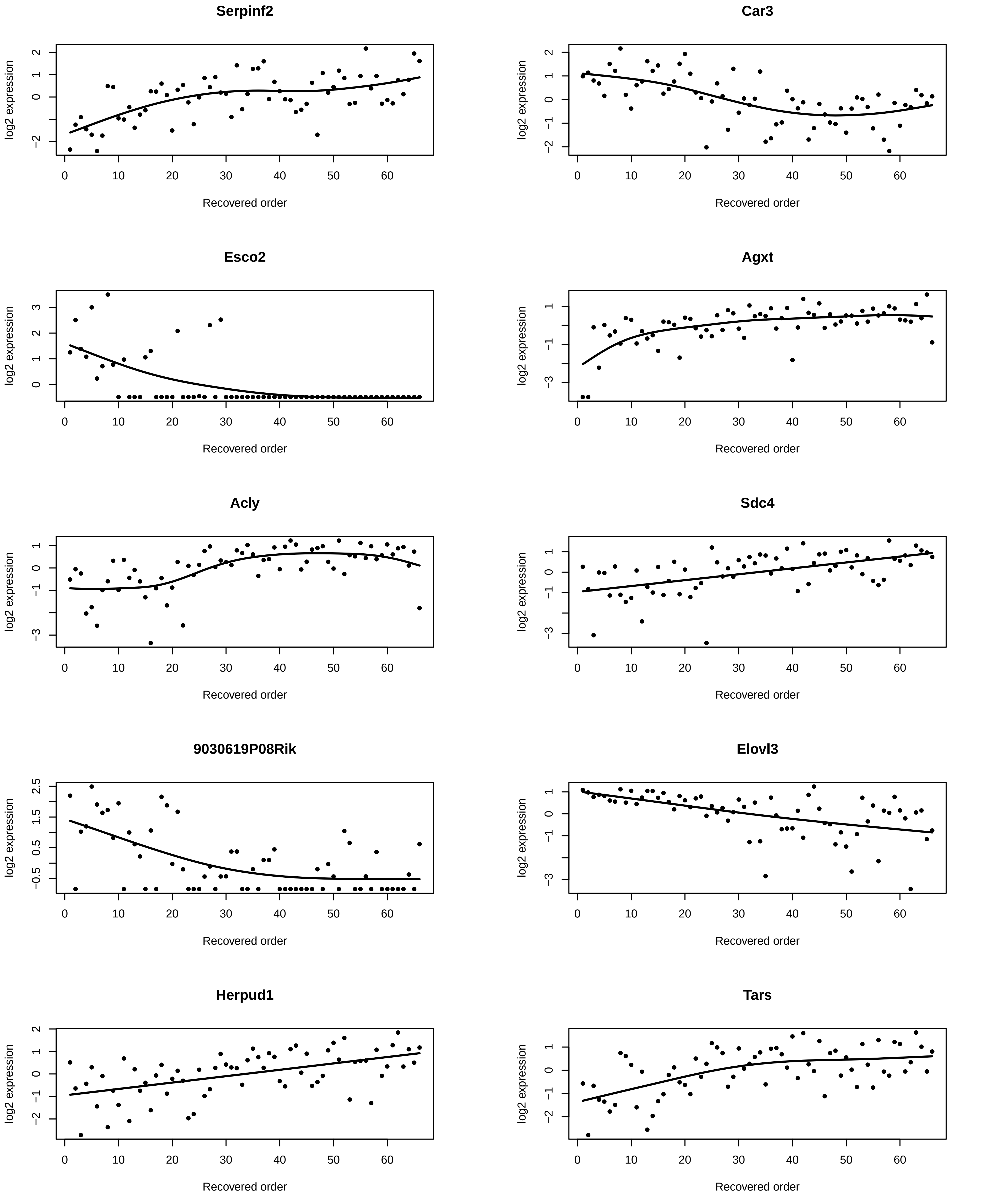
Serpinf2
Car3
Esco2
Agxt
Acly
Sdc4
9030619P08Rik
Elovl3
Herpud1
Tars
log2 expression
Recovered order

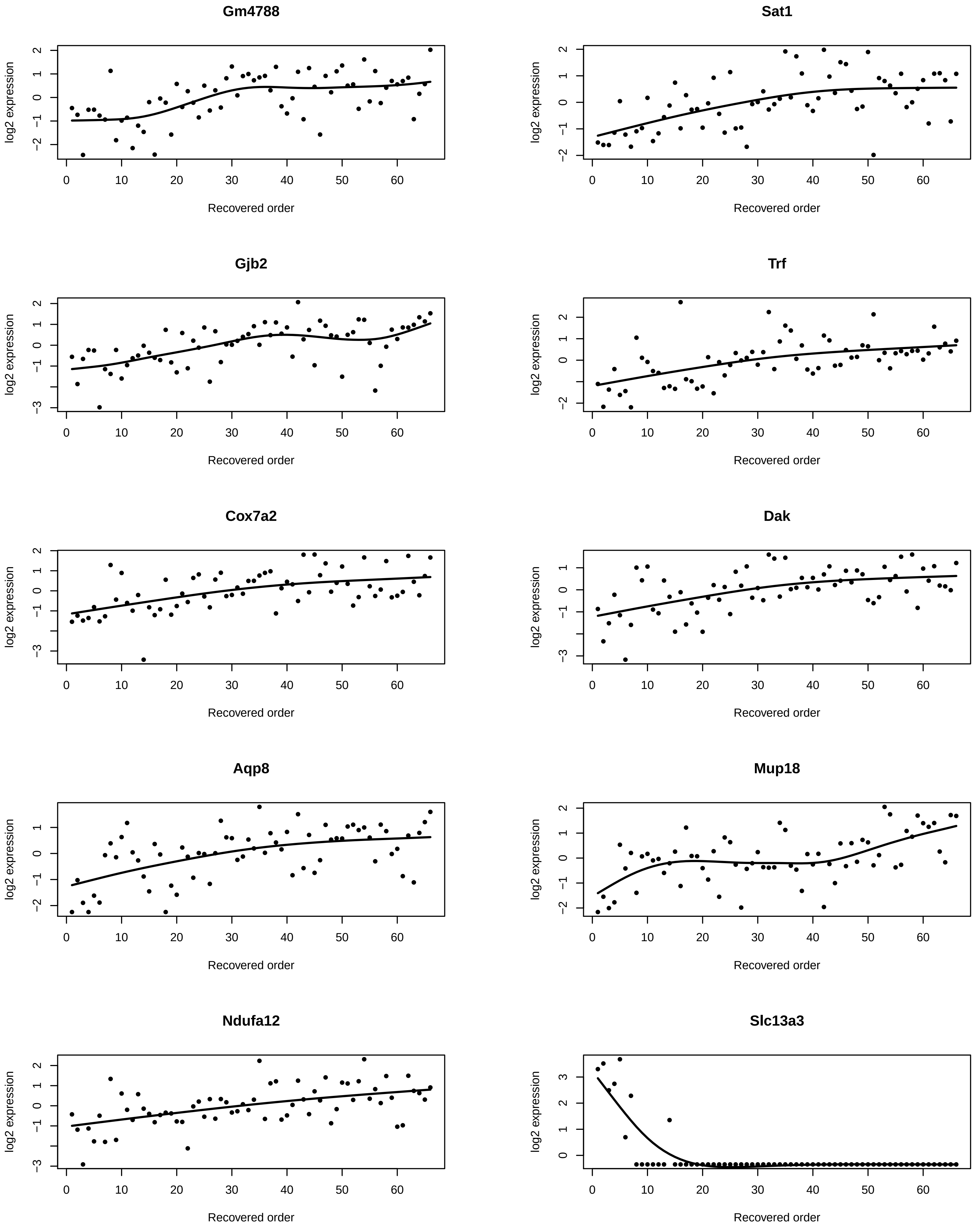
Gm4788
Sat1
Gjb2
Trf
Cox7a2
Dak
Aqp8
Mup18
Ndufa12
Slc13a3
log2 expression
Recovered order
0
10
20
30
40
50
60

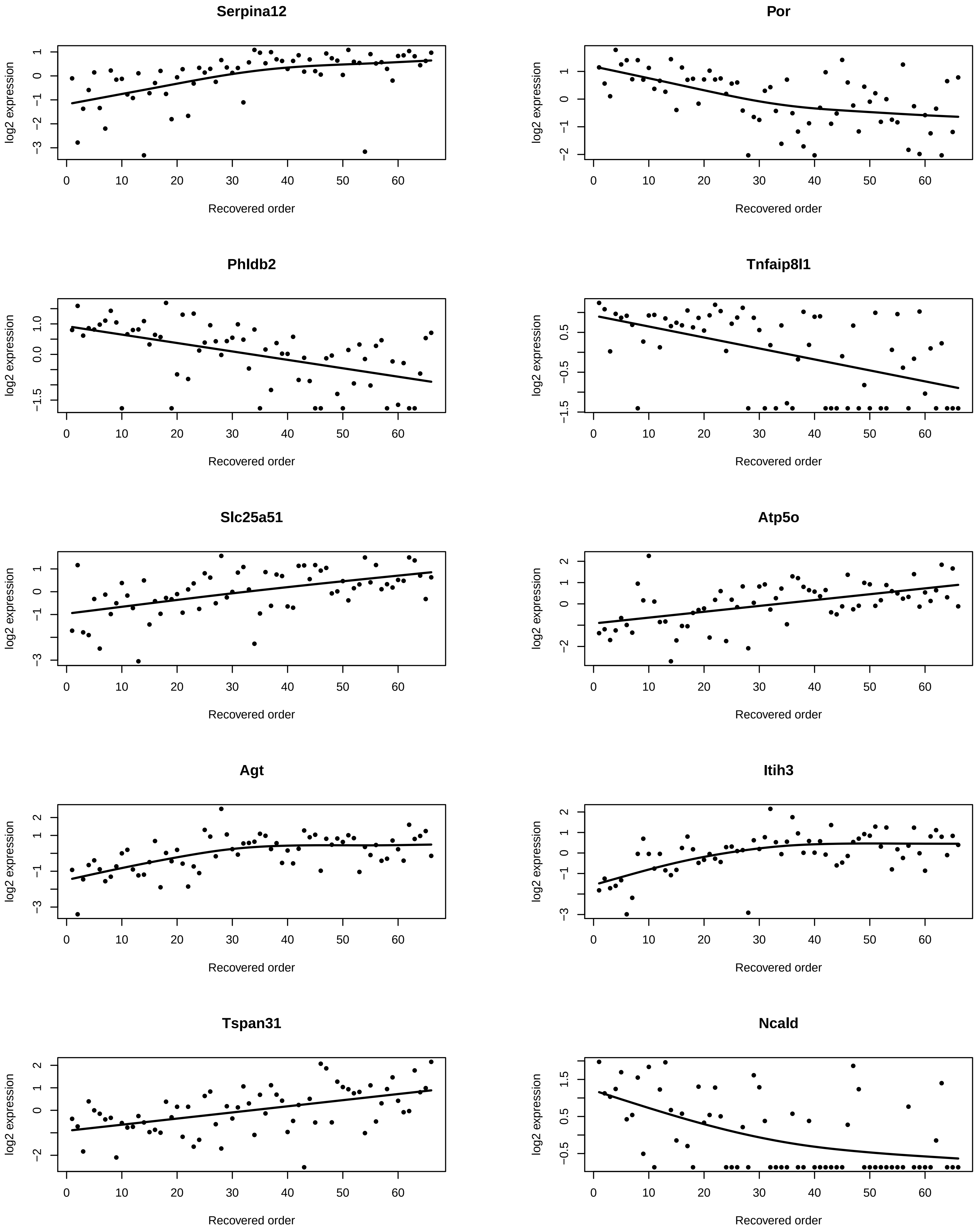
Serpina12
Por
Phldb2
Tnfaip8l1
Slc25a51
Atp5o
Agt
Itih3
Tspan31
Ncald
log2 expression
Recovered order

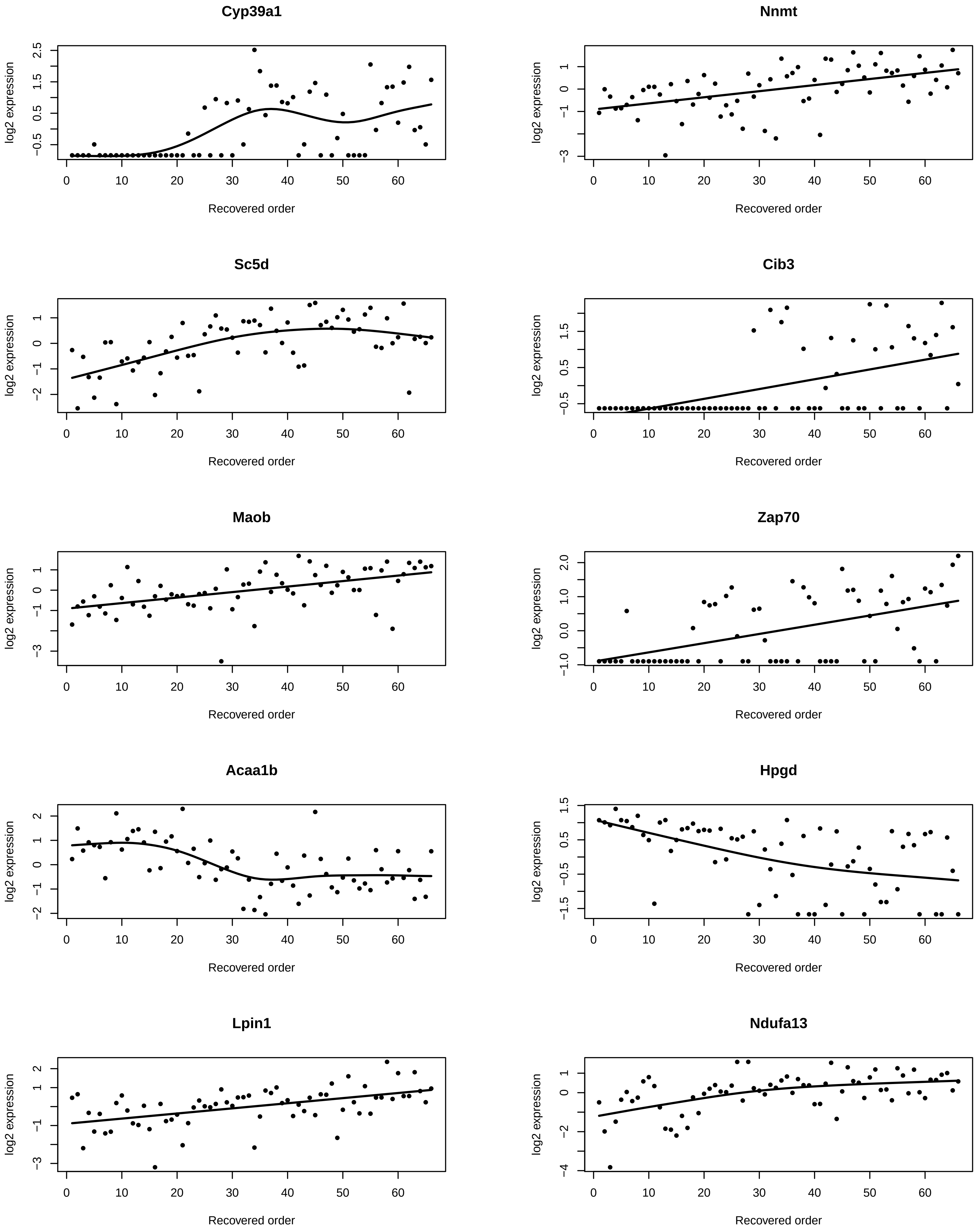
Cyp39a1
Nnmt
Sc5d
Cib3
Maob
Zap70
Acaa1b
Hpgd
Lpin1
Ndufa13
log2 expression
Recovered order

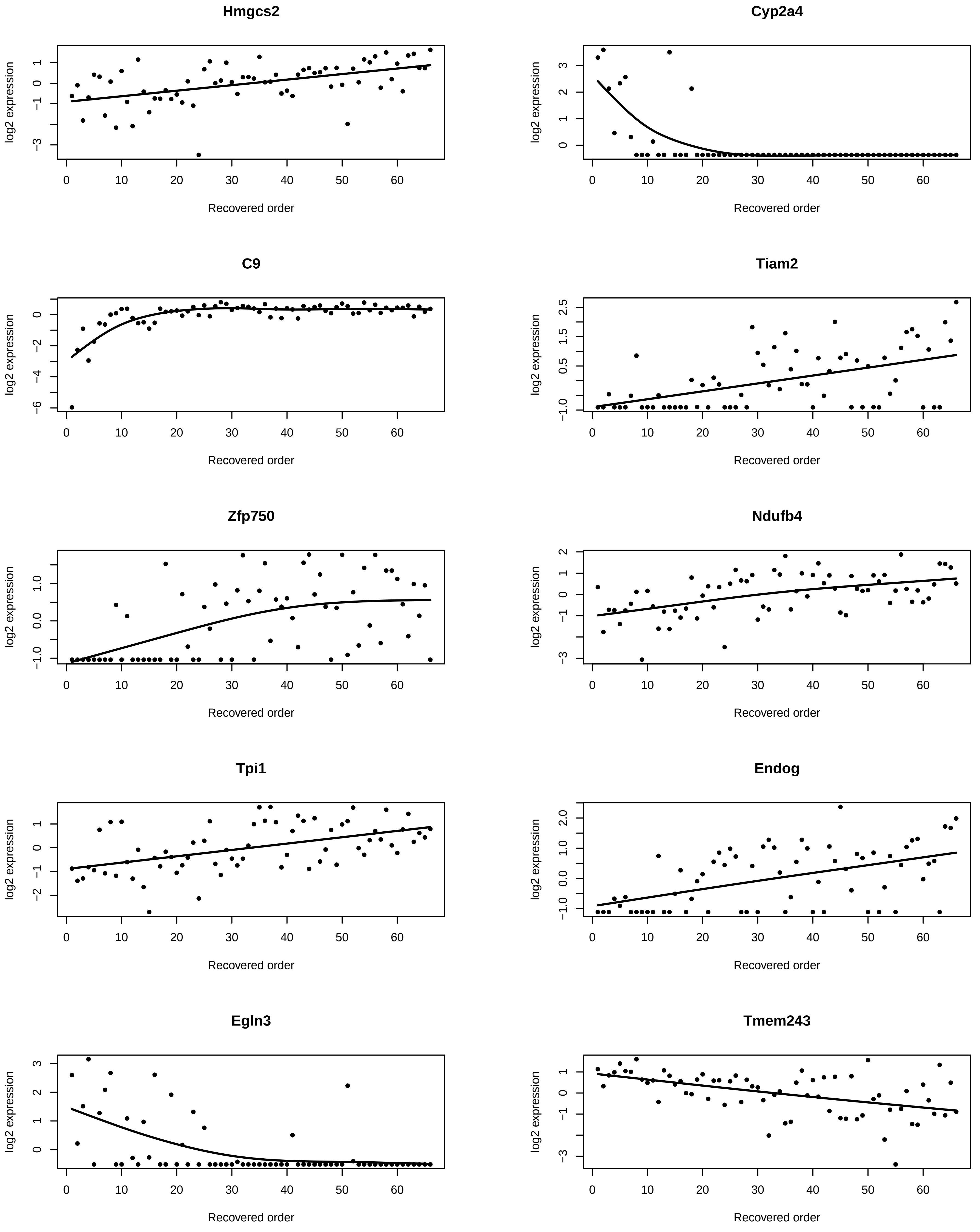
Hmgcs2
Cyp2a4
C9
Tiam2
Zfp750
Ndufb4
Tpi1
Endog
Egln3
Tmem243
log2 expression
Recovered order

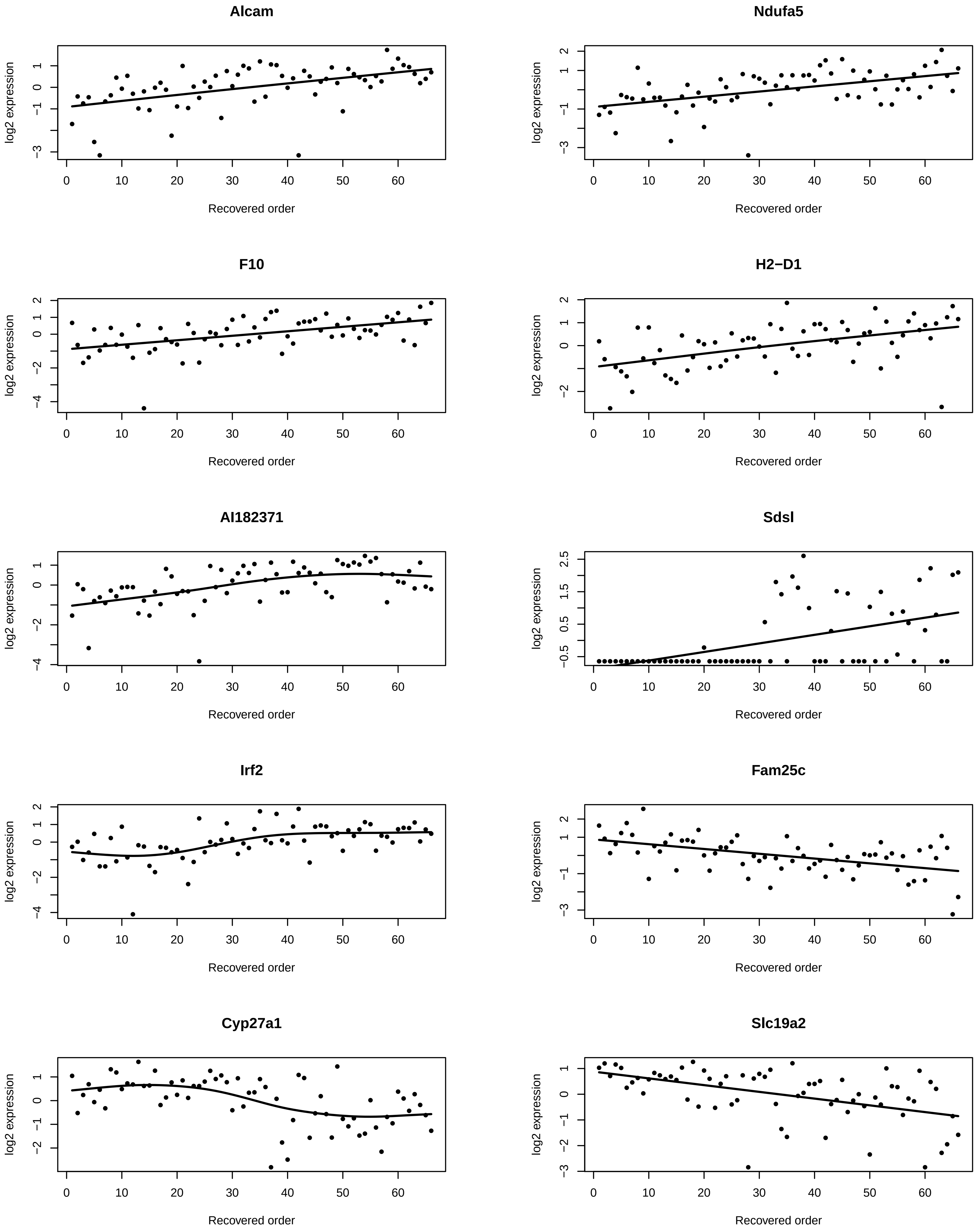
Alcam
Ndufa5
F10
H2−D1
AI182371
Sdsl
Irf2
Fam25c
Cyp27a1
Slc19a2
log2 expression
Recovered order

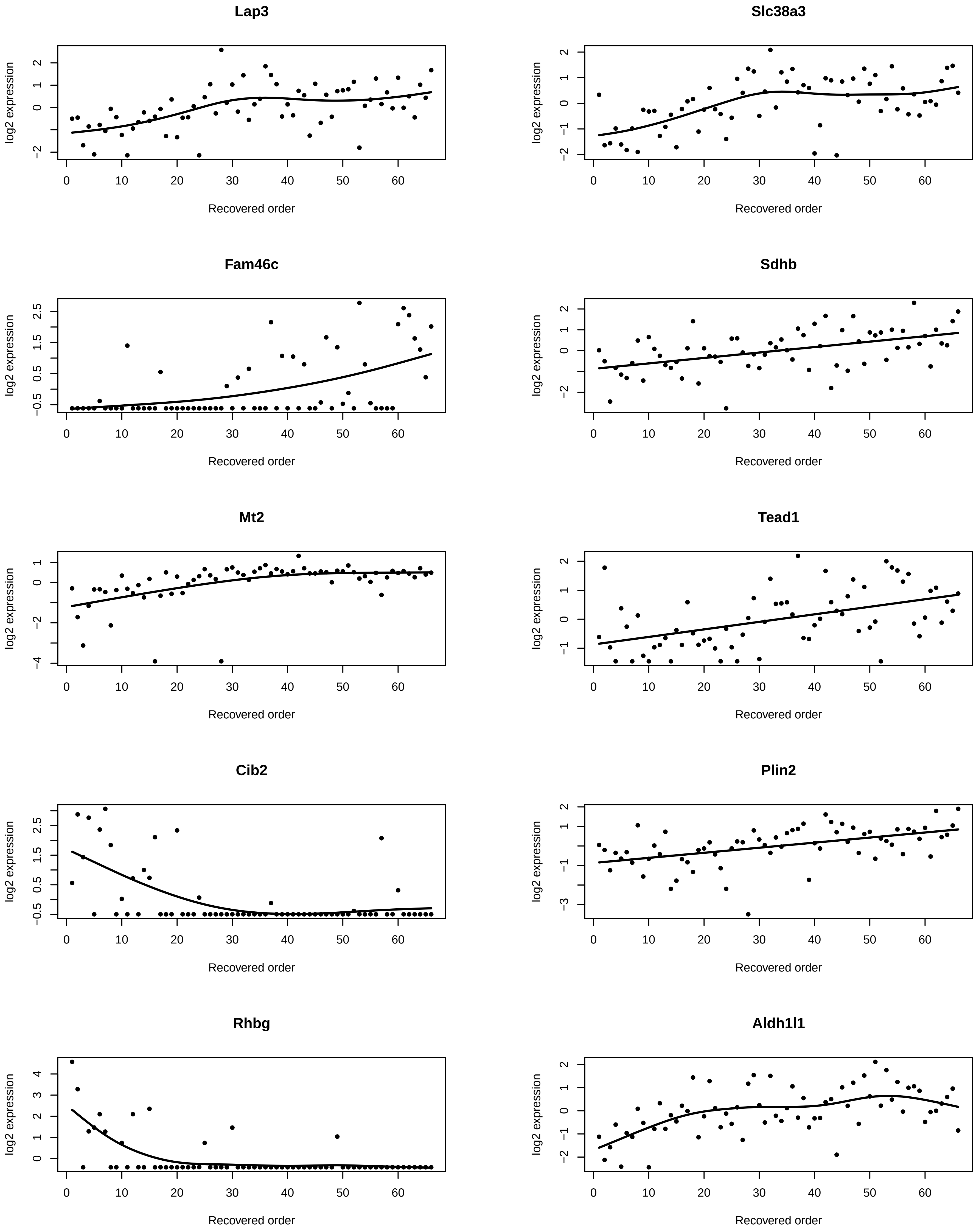

Lap3
Slc38a3
Fam46c
Sdhb
Mt2
Tead1
Cib2
Plin2
Rhbg
Aldh1l1
log2 expression
Recovered order

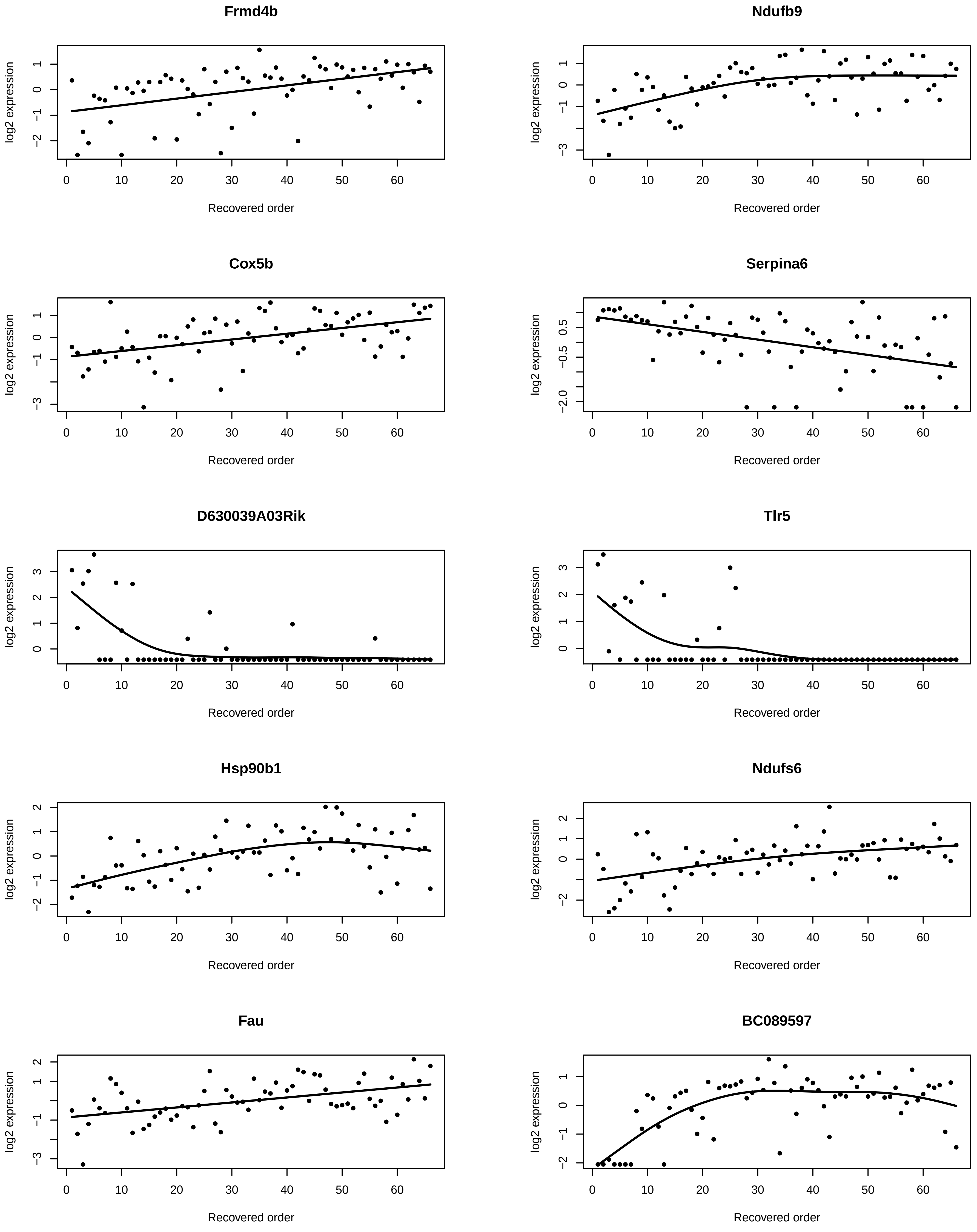

Frmd4b
Ndufb9
Cox5b
Serpina6
D630039A03Rik
Tlr5
Hsp90b1
Ndufs6
Fau
BC089597
log2 expression
Recovered order

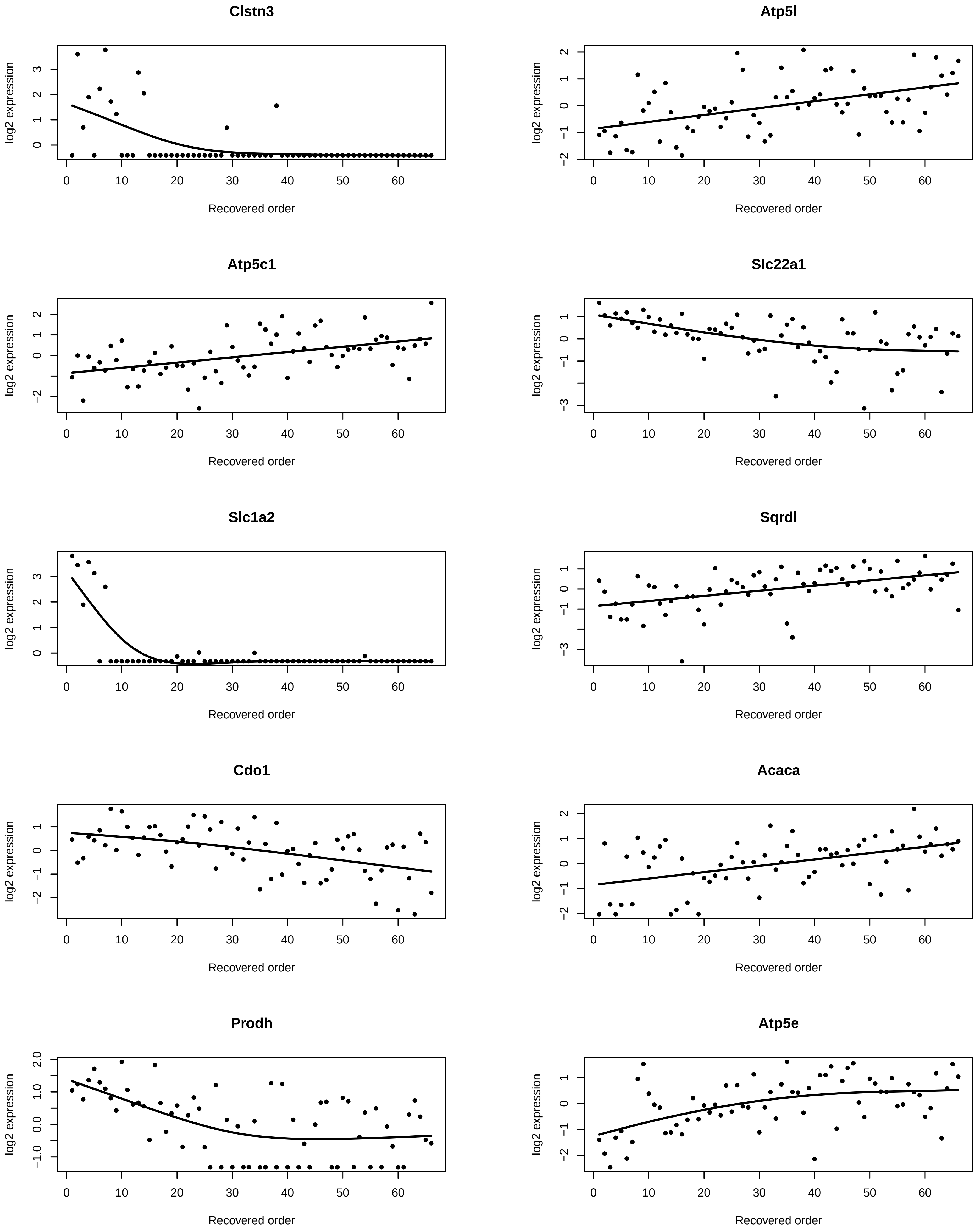

Clstn3
Atp5l
Atp5c1
Slc22a1
Slc1a2
Sqrdl
Cdo1
Acaca
Prodh
Atp5e
log2 expression
Recovered order

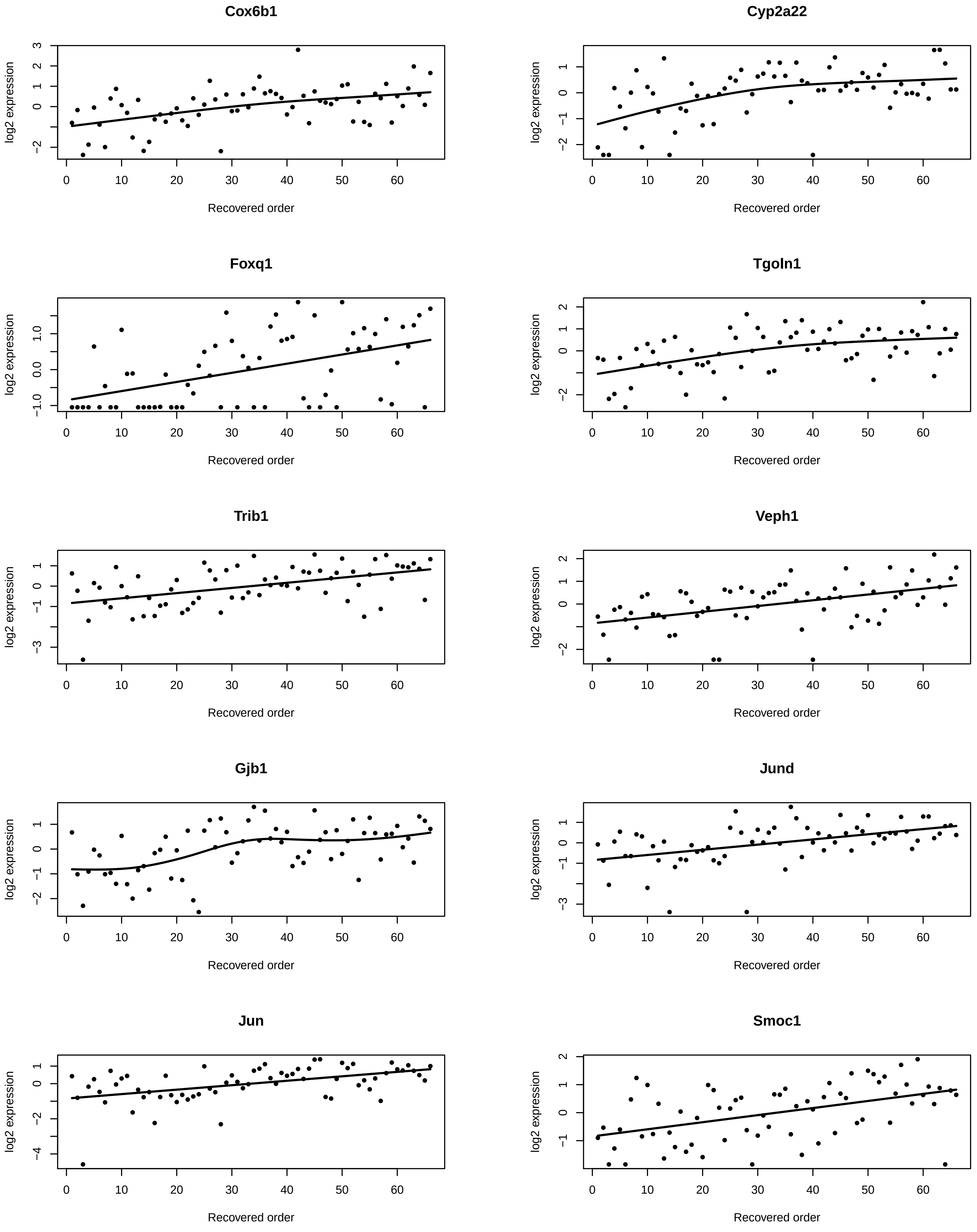
Cox6b1
Cyp2a22
Foxq1
Tgoln1
Trib1
Veph1
Gjb1
Jund
Jun
Smoc1
log2 expression
Recovered order
0
10
20
30
40
50
60

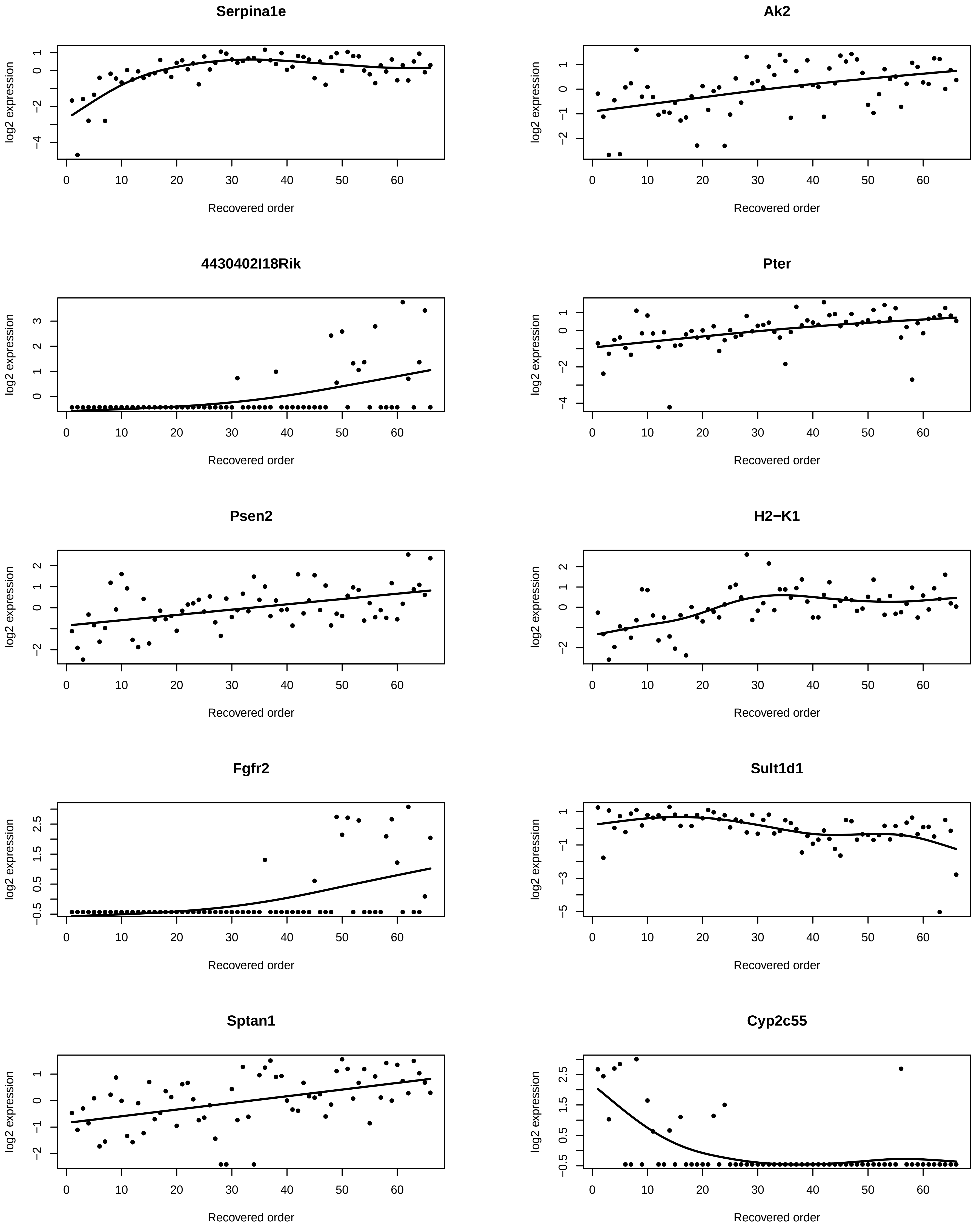
Serpina1e
Ak2
4430402I18Rik
Pter
Psen2
H2−K1
Fgfr2
Sult1d1
Sptan1
Cyp2c55
log2 expression
Recovered order

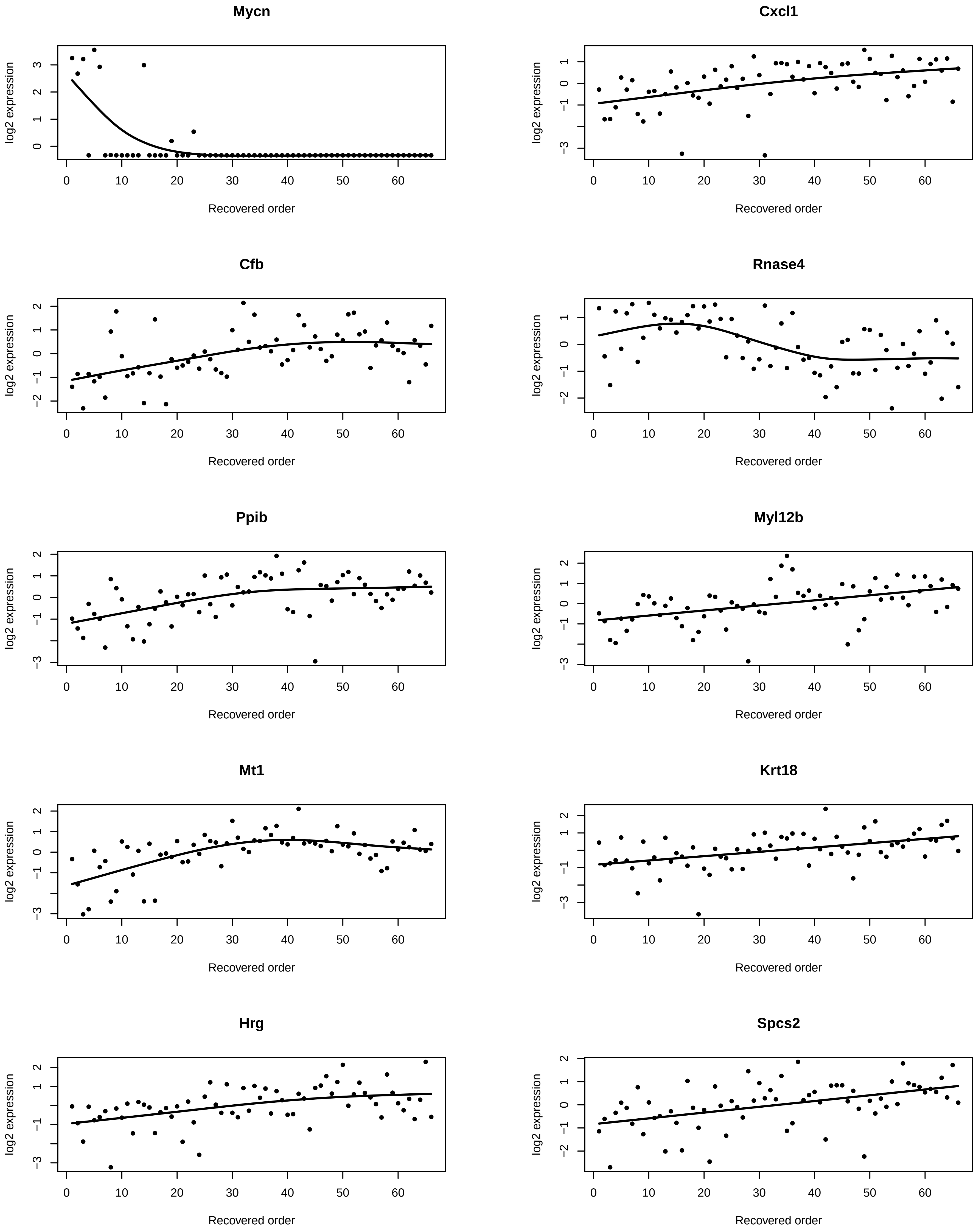
Mycn
Cxcl1
Cfb
Rnase4
Ppib
Myl12b
Mt1
Krt18
Hrg
Spcs2
log2 expression
Recovered order

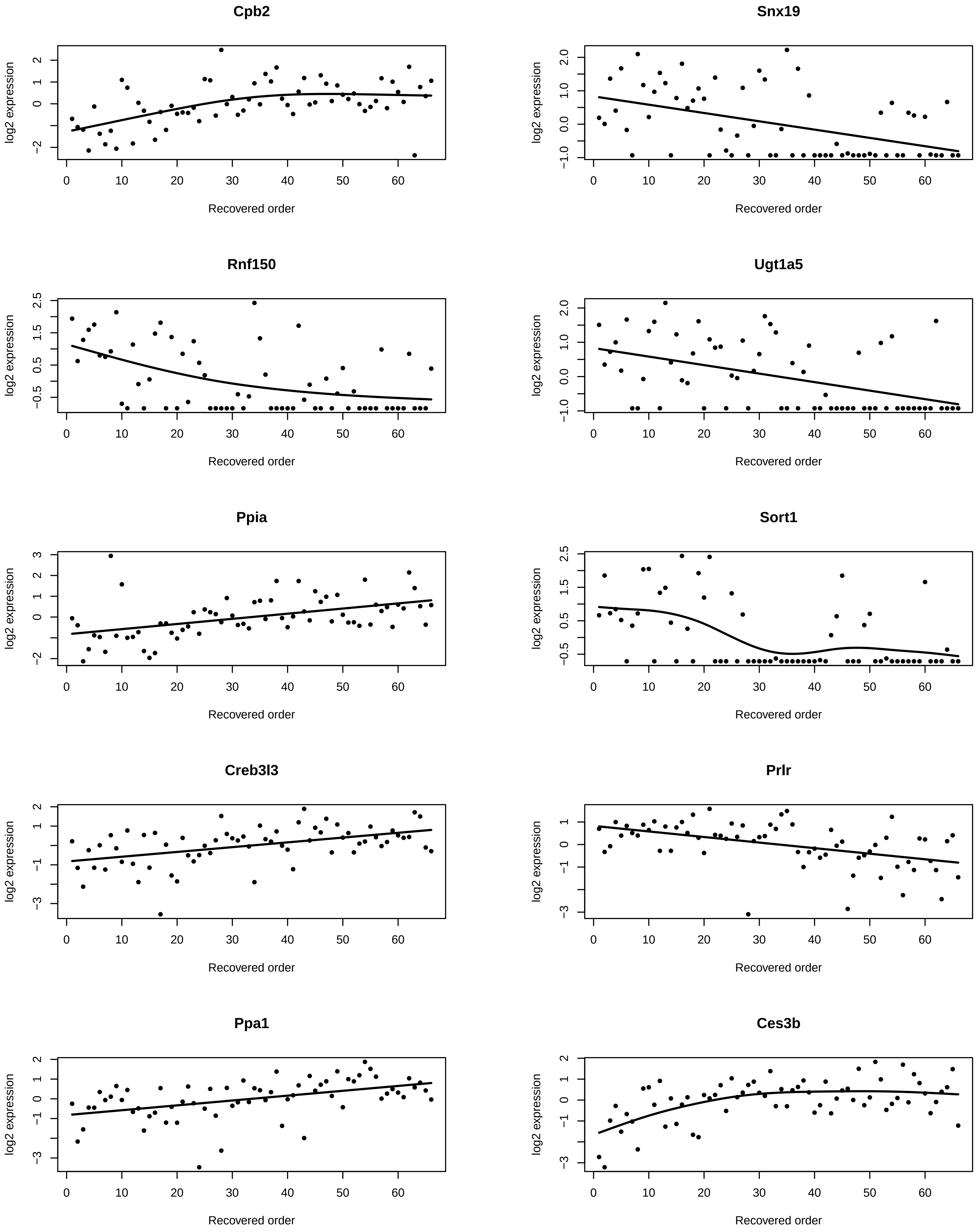
Cpb2
Snx19
Rnf150
Ugt1a5
Ppia
Sort1
Creb3l3
Prlr
Ppa1
Ces3b
log2 expression
Recovered order

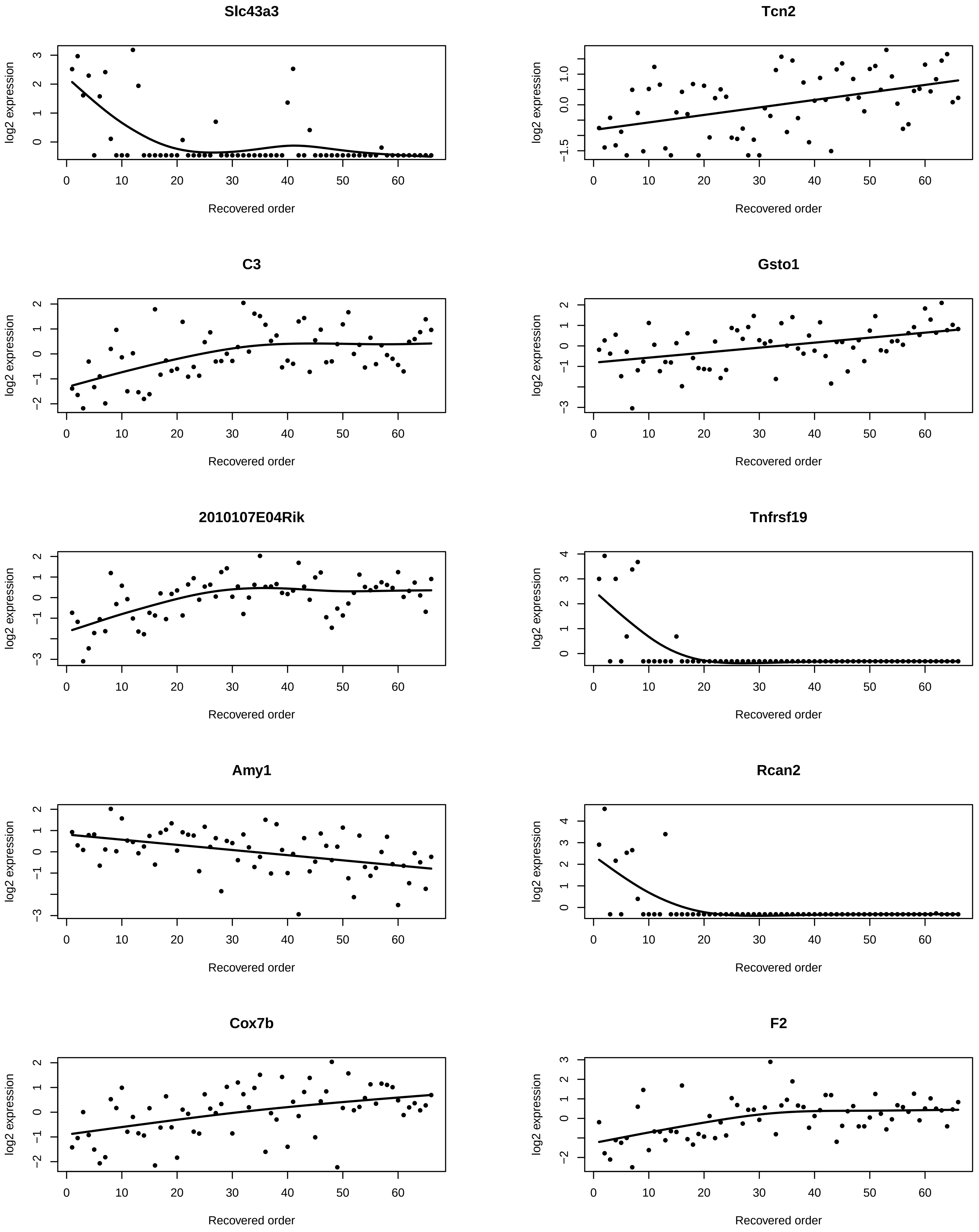
Slc43a3
Tcn2
C3
Gsto1
2010107E04Rik
Tnfrsf19
Amy1
Rcan2
Cox7b
F2
log2 expression
Recovered order

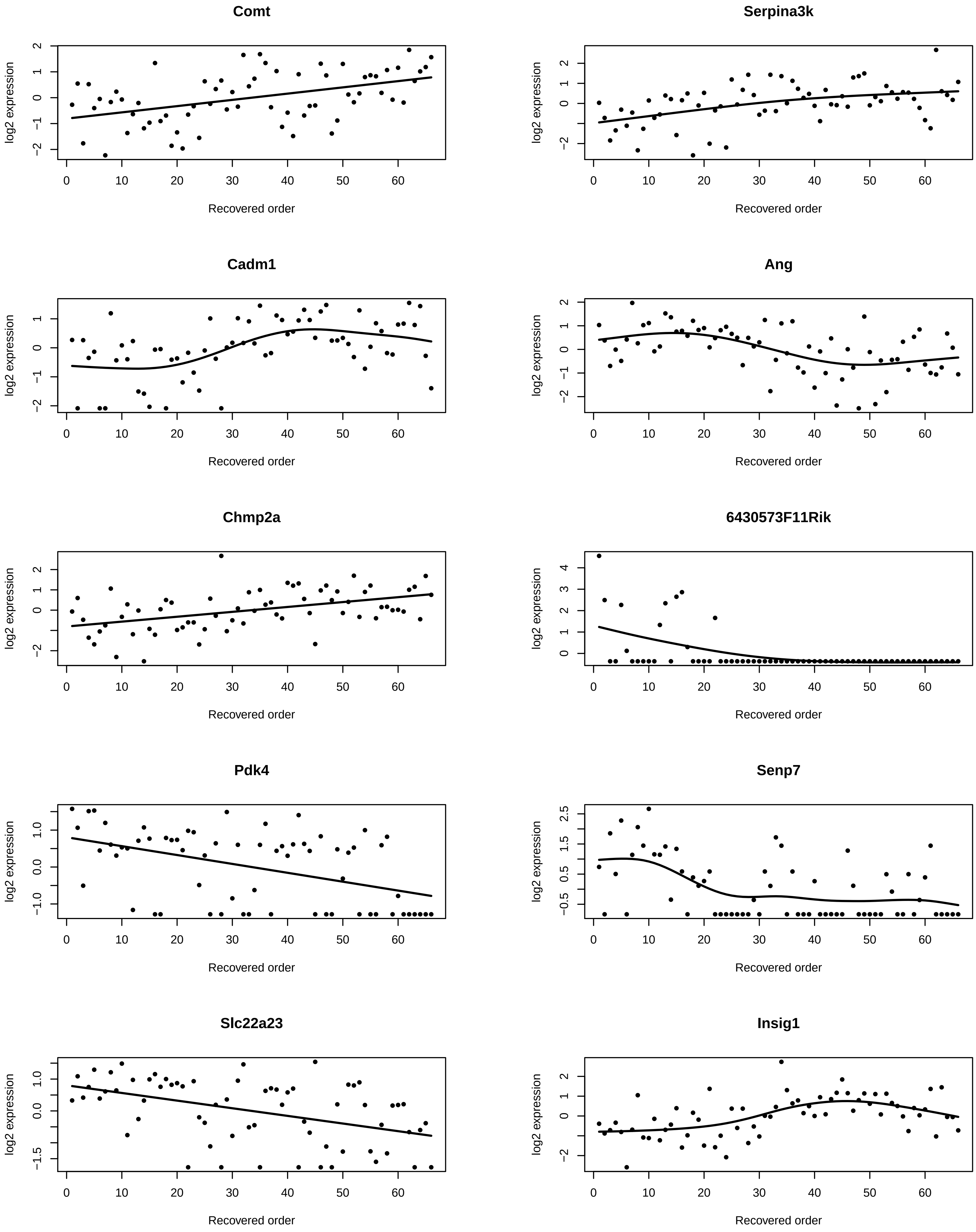
Comt
Serpina3k
Cadm1
Ang
Chmp2a
6430573F11Rik
Pdk4
Senp7
Slc22a23
Insig1
log2 expression
Recovered order

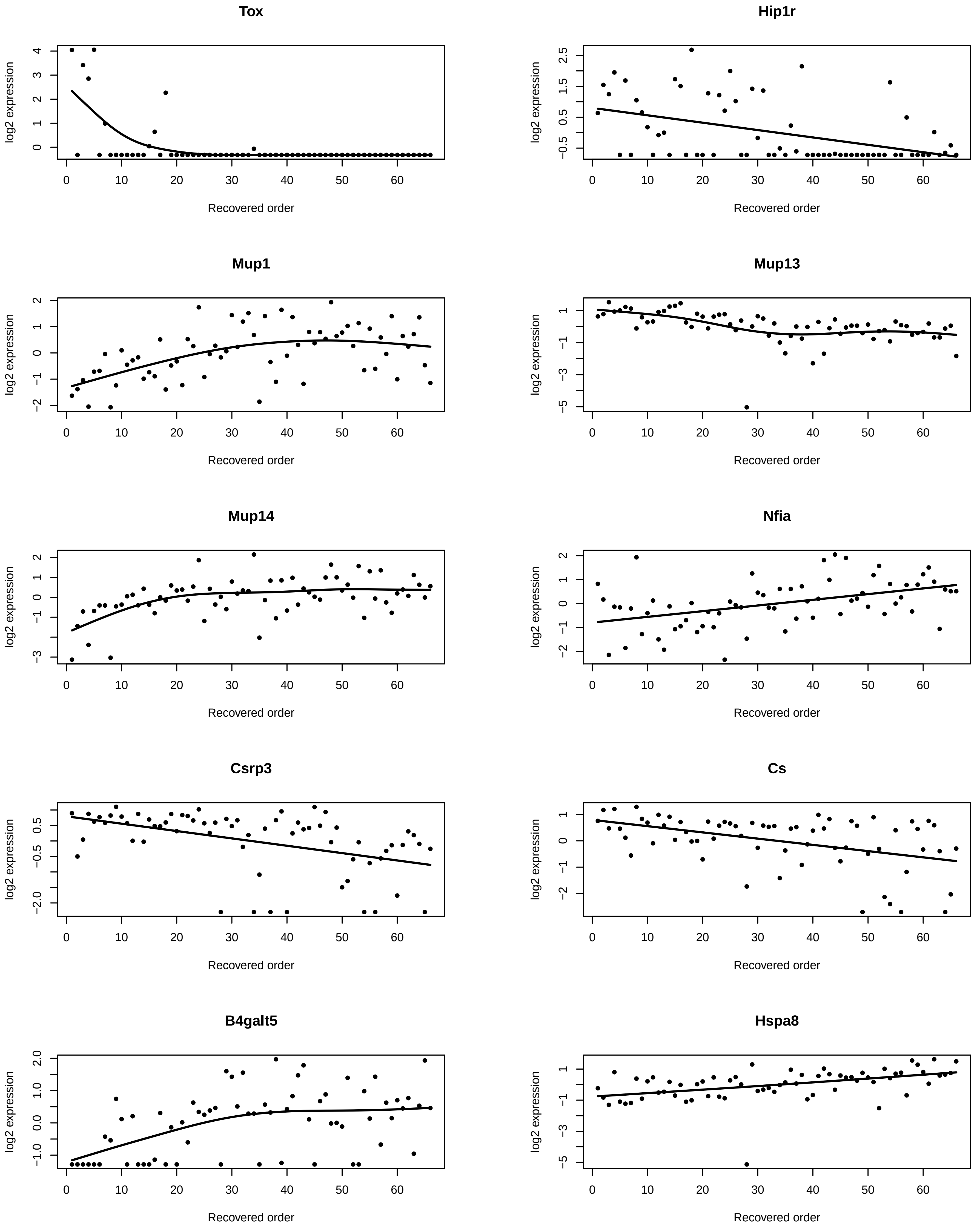
Tox
Hip1r
Mup1
Mup13
Mup14
Nfia
Csrp3
Cs
B4galt5
Hspa8
log2 expression
Recovered order

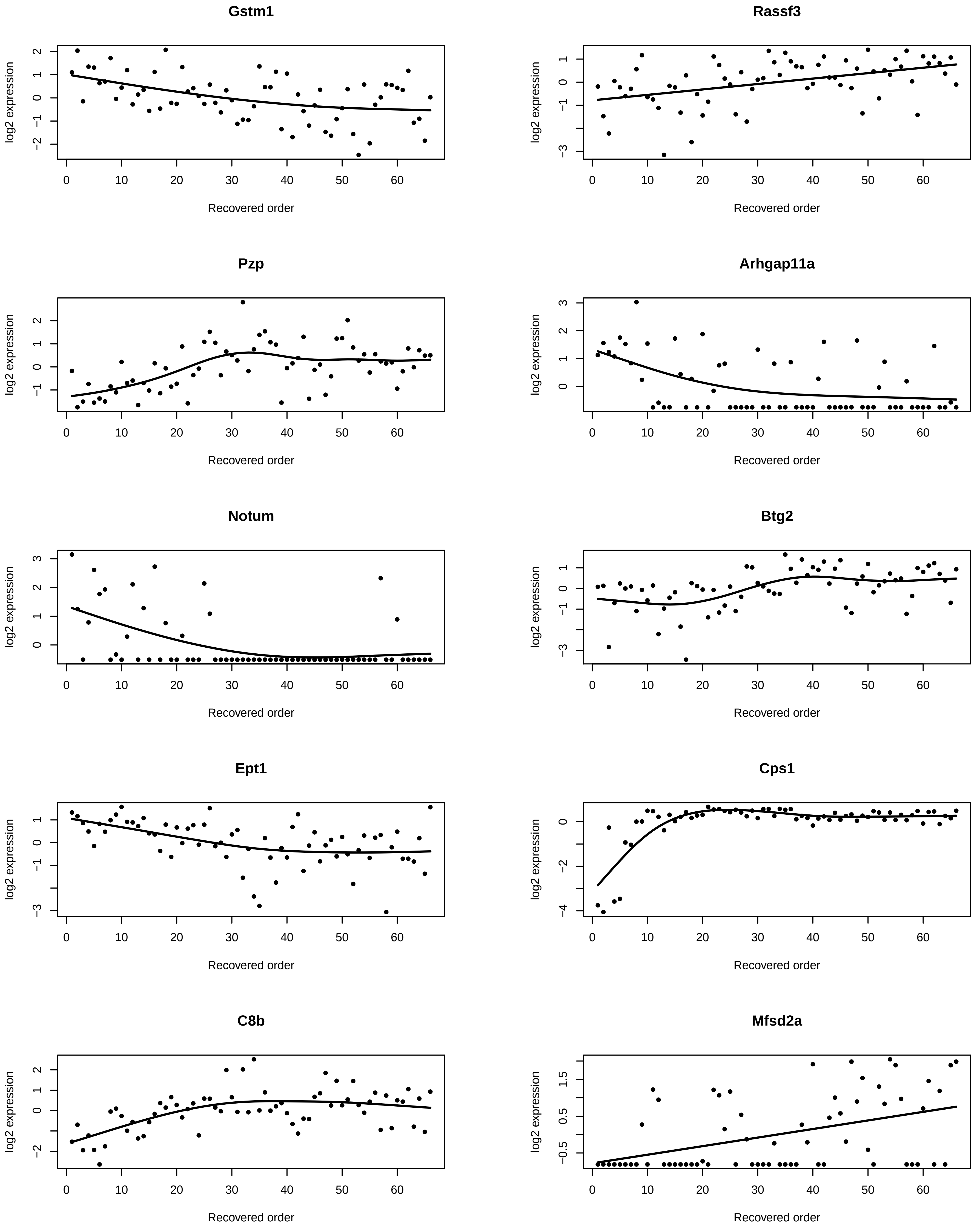

Gstm1
Rassf3
Pzp
Arhgap11a
Notum
Btg2
Ept1
Cps1
C8b
Mfsd2a
log2 expression
Recovered order

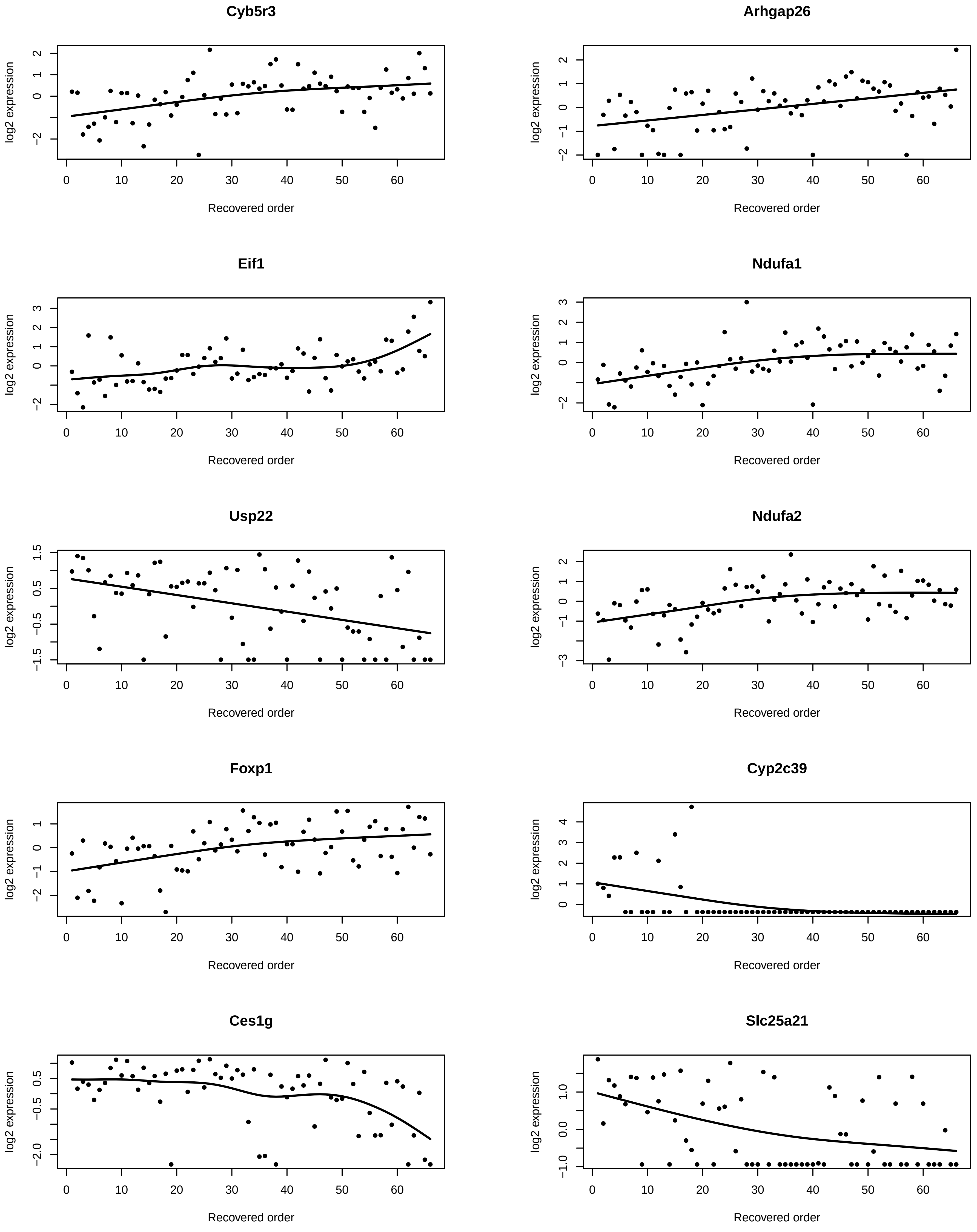
Cyb5r3
Arhgap26
Eif1
Ndufa1
Usp22
Ndufa2
Foxp1
Cyp2c39
Ces1g
Slc25a21
log2 expression
Recovered order

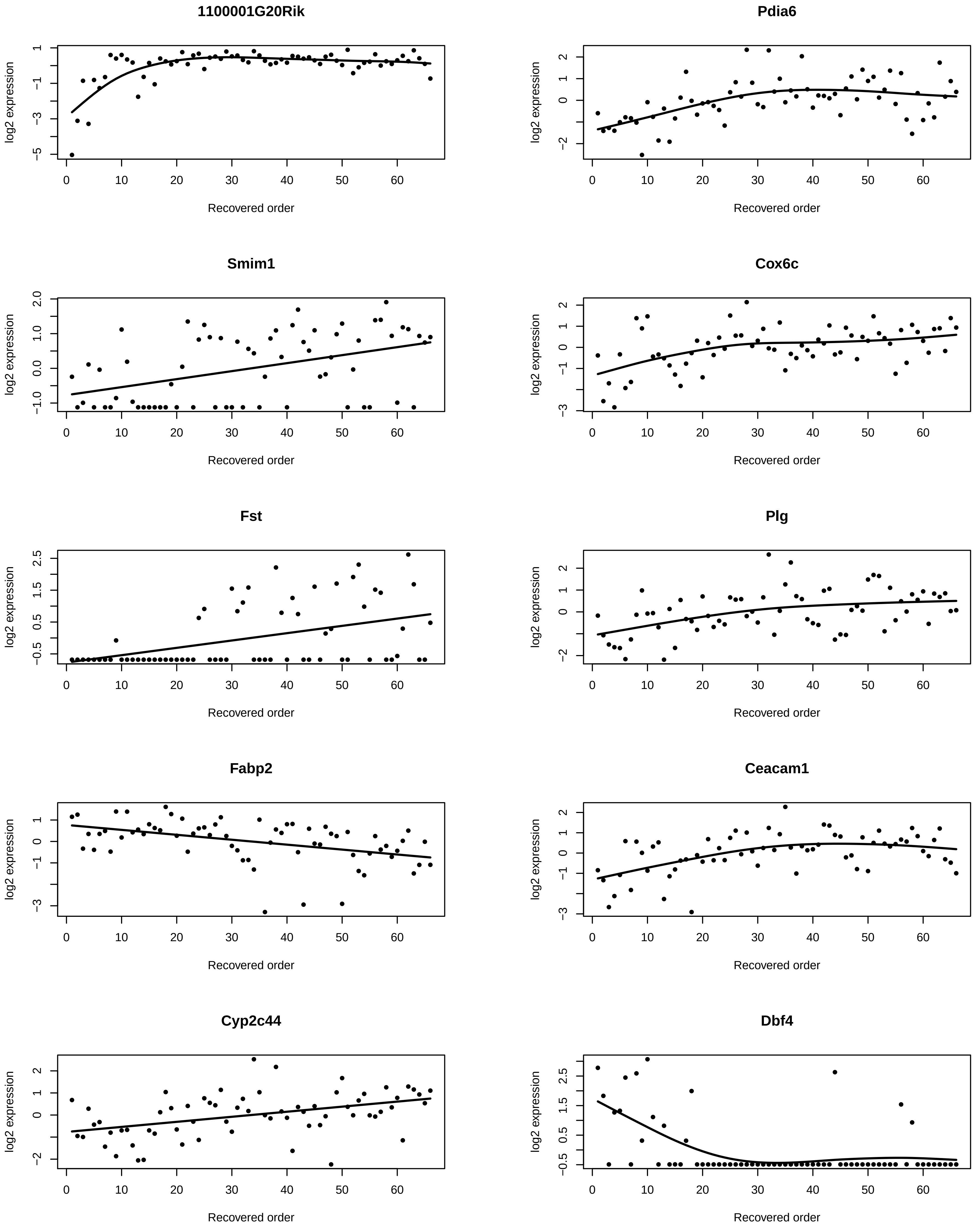

1100001G20Rik
Pdia6
Smim1
Cox6c
Fst
Plg
Fabp2
Ceacam1
Cyp2c44
Dbf4
log2 expression
Recovered order
0
10
20
30
40
50
60

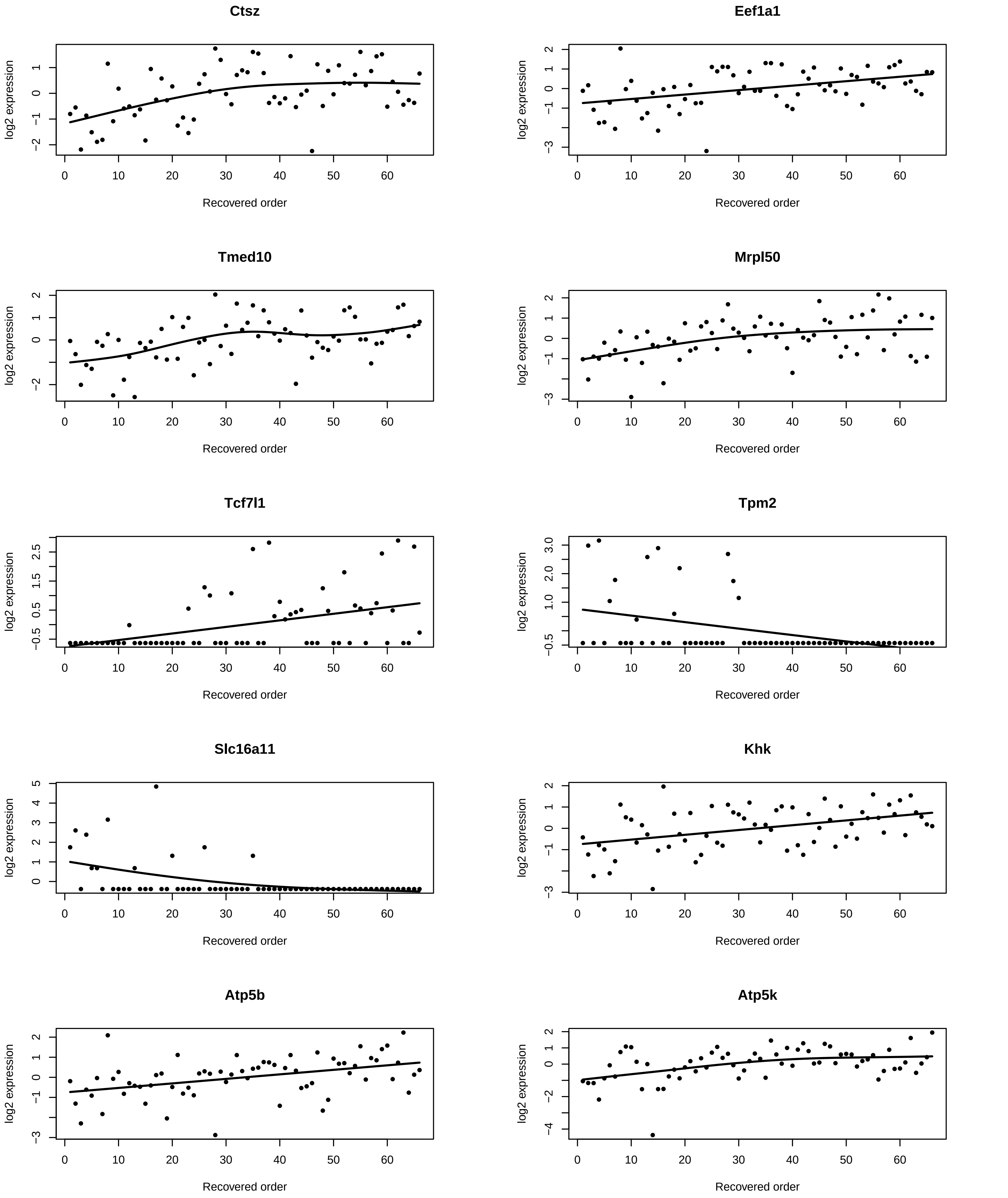
Ctsz
Eef1a1
Tmed10
Mrpl50
Tcf7l1
Tpm2
Slc16a11
Khk
Atp5b
Atp5k
log2 expression
Recovered order

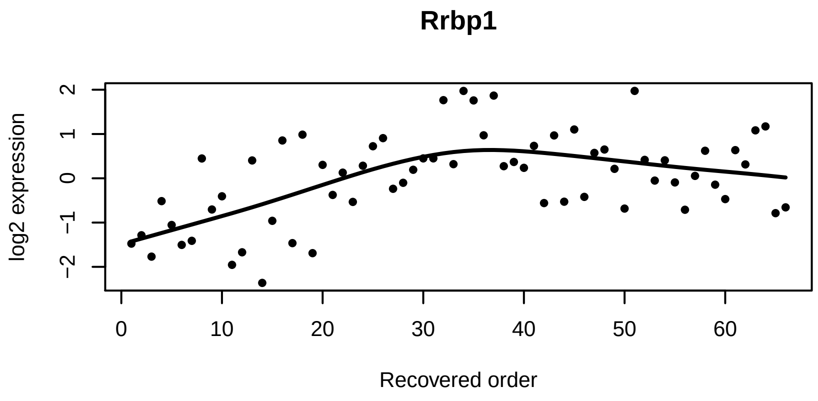
Rrbp1
log2 expression
−2
−1
0
1
2
0
10
20
30
40
50
60
Recovered order

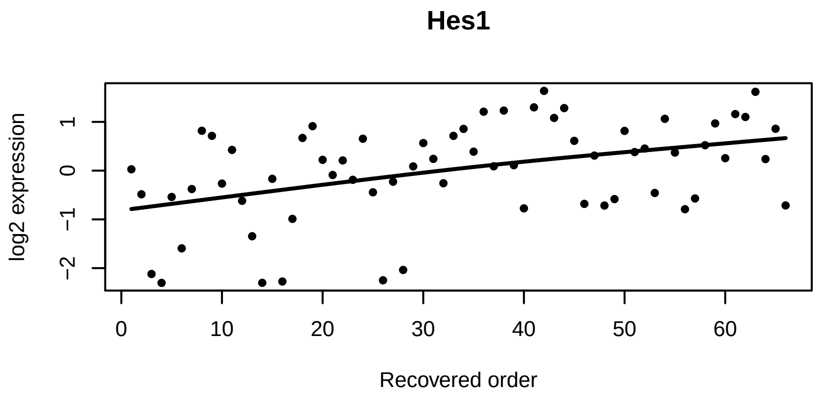
Hes1
log2 expression
−2
−1
0
1
0
10
20
30
40
50
60
Recovered order
