# Supplementary material for: Reproducibility across single-cell RNA-seq protocols for spatial ordering analysis
Source: PLoS One. 2020 Sep 28;15(9):e0239711. doi: 10.1371/journal.pone.0239711 (PMC7521718; doi:10.1371/journal.pone.0239711)
Supplement: Attachment — Submitted filename: Response_to_Reviewers.docx [file pone.0239711.s011.docx]

**Response to Reviewers**

**Reviewer #1:**

**1 The logic of this paper is unclear. It should has been concentrated on the comparison of different protocols and evaluation of alternatives.**

We apologize that our focus on the comparison of the protocols was not more clear. We have made a number of changes to the manuscript to improve clarity. Specifically:

-We added additional analysis and removed extraneous details to the section on the enrichment analysis. We narrowed the focus to genes that are significantly zonated in all three datasets to align with our goal of comparing the protocols. We hope the logic of this section is much clearer.

“We expanded our analysis to identify additional dynamic genes, those with significant differential expression along the reconstructed spatial order, by modeling gene expression as a function of the reconstructed zonation axis (Methods). Genes that were ‘significantly zonated’ in all datasets (adjusted p-value < .1) had highly correlated expression profiles. The Smart-seq versus MARS-seq expression profiles had the highest median correlation (0.86), while Smart-seq versus 10X had the lowest median correlation (0.69). The significantly zonated genes shared by all three datasets (Figure 4A) were enriched in KEGG metabolic processes with known periportal or pericentral bias such as amino acid metabolism (periportal), retinol metabolism (pericentral), and CYP450 metabolism (pericentral). Among the significantly zonated genes in the broad “Metabolic pathways” category in KEGG, the median correlation between all datasets ranged from 0.75 to 0.89 (Figure 4B). When all genes were considered the median correlation ranged from 0–0.04. A handful of genes were significantly zonated in all datasets but had low correlation in expression profiles (S4 Figure). We found these genes were generally influenced by additional factors such as the circadian clock or diet (e.g. Insig227, Mt1 and Mt228, Scp229, and Mup230) or sex (e.g. Apoc131). Despite using different reordering algorithms and protocols, the three datasets show high agreement of expression along the recovered pericentral to periportal axis among genes that are significantly zonated in all datasets, and reliably mirror the in vivo patterning of the liver lobule (S5 Figure).”

-We have also re-written a number of sentences to improve clarity. A full accounting of these changes is highlighted in the tracked manuscript.

**2 The statistical analysis is not intuitional.**

We apologize the analyses were not explained in a logical way. We have added additional text to explain the purpose and relevance of each analysis. Specifically:

-“Next, we reconstructed the spatial organization across the liver lobule by computationally ordering the cells in the three datasets according to their expression profiles.”

-“Since the MARS-seq dataset placed cells into nine discrete zones along the axis, we also divided the ordered cells from the Smart-seq and 10X datasets into nine equally sized groups in order to directly compare the gene expression profiles. The initial zonation groups are aligned with the pericentral region and the later zonation groups align with the periportal region.”

-“We expanded our analysis to identify additional dynamic genes, those with significant differential expression along the reconstructed spatial order, by modeling gene expression as a function of the reconstructed zonation axis (Methods).”

-“Beyond gene-level expression dynamics, we also evaluated isoform analysis – another exciting field of study with scRNA-seq data.”

-“In a further comparison of the protocols we performed a subsampling experiment to study the trade-offs between higher depth versus more cells.”

In addition, we have removed the k-means analysis as it was not directly relevant or insightful to the comparison of the three protocols.

**3 Incorrect subscripts, inconsistent formatting and inappropriate subtitles alongside causing ambiguity.**

We apologize for the confusion. We have made sure to stick to the pericentral and periportal phrasings and only use PC and PP as abbreviations in the figures where necessary. We have also defined how the zonation groups correspond to the pericentral to periportal axis in the text:

“The initial zonation groups are aligned with the pericentral region and the later zonation groups align with the periportal region.”

We also more clearly define ‘significantly zonated’ as those genes displaying significant dynamic expression across the zonation groups. “We expanded our analysis to identify additional dynamic genes, those with significant differential expression along the reconstructed spatial order, by modeling gene expression as a function along the reconstructed zonation axis (Methods). Genes that were ‘significantly zonated’ in all datasets (adjusted p-value < .1) had highly correlated expression profiles.”

All subtitles in the figures have been made to match the wording used in the text and defined clearly. A full accounting of these changes is highlighted in the tracked manuscript.

**4 The paper has not given alternative strategies, authors should hold further discussions.**

This is a great point. We have expanded the discussion to alternative methods that may be used for spatial profiling. Specifically, “We acknowledge that inferring spatial information in silico has limitations and requires extensive validation. Other methods are being developed that directly detect the spatial pattern of mRNA expression such as small molecule in-situ hybridization^34,35^ where mRNA molecules in tissue sections are directly labeled by complementary probes allowing visualization of the spatial location. Unfortunately, this approach is expensive, labor intensive, and low throughput. Another recently developed technique is spatial sequencing^36–39^ where a tissue slice is placed on top of an array of pre-barcoded oligo(dT) primers and imaged. The mRNA is then bound to the primers in the array and the original spatial location of an mRNA is subsequently inferred based on the sequenced barcode. While this method provides transcriptomic data at an unprecedented accuracy and high spatial resolution, the cells are sequenced at lower total depths and is limited to the detection of the 3’ end of the mRNA. The sampling technique is further prone to sample mRNA from more than one cell and thus lowers the transcriptomic resolution compared to single cell sequencing.”

**Reviewer #2: Single cell sequencing is getting more popular. In this manuscript, the authors compared the performance of smart-seq, which is deep sequencing and the MARS-seq, the shallower one. Their findings indicated useful hints for choosing the protocol depending on the research purpose. It is also interesting to know that smart-seq generally identified more genes and agrees with the majority of genes identified using the other two methods. The manuscript is well written and is also a good learning material. It is not difficult to understand with the well-presented figures. I only get one question. Gene Cyp8b1 indicated flat curve using smart-seq method, which is different with MARS-seq results. Does it mean that the gene expression scale depends on the method applied? If so, why only one gene act like this, not the other genes? Generally, I think the manuscript indicated interesting results and shows the privilege of using Smart-seq, I would advise the manuscript to be accepted.**

Although the smooth fit of Cyp8b1 appears flat, the shape follows that of 10X with the effect dampened due to high variability in the group medians (Group 4 and 6 specifically). We rephrased this in the manuscript to “Cyp8b1 is found to have flatter expression levels along most of the lobule”. Interestingly, this gene has been shown to vary based on feeding time and the circadian clock^1^, thus some variability may be attributed to uncontrollable factors across experiments. We appreciate this comment and expanded our analysis to identify genes that are significantly zonated in all three datasets, yet have low correlations in their expression profiles. In the boxplot below, all pairwise correlations for significantly zonated genes are shown. The vast majority are highly correlated. However, there are a handful with low correlation. We find a large number of these genes are associated with additional variables including circadian cycle, sex, and diet. Specifically, “We found these genes were generally influenced by additional factors such as the circadian clock or diet (e.g. Insig227, Mt1 and Mt228, Scp229, and Mup230) or sex (e.g. Apoc131).” We have added this analysis to the manuscript and in S4 Figure.

References

1. Eggink HM, Oosterman JE, de Goede P, de Vries EM, Foppen E, Koehorst M, et al. Complex interaction between circadian rhythm and diet on bile acid homeostasis in male rats. Chronobiology International. 2017 Nov 26;34(10):1339–53.
